# Removal of Barium from Solution by Natural and Iron(III) Oxide-Modified Allophane, Beidellite and Zeolite Adsorbents

**DOI:** 10.3390/ma13112582

**Published:** 2020-06-05

**Authors:** Andre Baldermann, Yvonne Fleischhacker, Silke Schmidthaler, Katharina Wester, Manfred Nachtnebel, Stefanie Eichinger

**Affiliations:** 1 Institute of Applied Geosciences & NAWI Graz Geocenter, Graz University of Technology, Rechbauerstraße 12, 8010 Graz, Austria; y.fleischhacker@stud.uni-graz.at (Y.F.); silke.schmidthaler@gmx.at (S.S.); katharina.wester@gmx.at (K.W.); stefanie.eichinger@tugraz.at (S.E.); 2 Institute of Electron Microscopy and Nanoanalysis, Graz Centre for Electron Microscopy (FELMI-ZFE), Steyrergasse 17, 8010 Graz, Austria; manfred.nachtnebel@felmi-zfe.at

**Keywords:** barium, adsorption, environment, wastewater treatment, surface modification

## Abstract

Efficient capture of barium (Ba) from solution is a serious task in environmental protection and remediation. Herein, the capacity and the mechanism of Ba adsorption by natural and iron(III) oxide (FeO) modified allophane (ALO), beidellite (BEI) and zeolite (ZEO) were investigated by considering the effects of contact time, temperature, pH, Ba^2+^ concentration, adsorbent dosage, the presence of competitive ions and adsorption–desorption cycles (regenerability). Physicochemical and mineralogical properties of the adsorbents were characterized by XRD, FTIR, SEM with EDX and N_2_ physisorption techniques. The Ba^2+^ adsorption fitted to a pseudo-first-order reaction kinetics, where equilibrium conditions were reached within <30 min. BEI, ALO and ZEO with(out) FeO-modification yielded removal efficiencies for Ba^2+^ of up to 99.9%, 97% and 22% at optimum pH (pH 7.5–8.0). Adsorption isotherms fitted to the Langmuir model, which revealed the highest adsorption capacities for BEI and FeO-BEI (44.8 mg/g and 38.6 mg/g at 313 K). Preferential ion uptake followed in the order: Ba^2+^ > K^+^ > Ca^2+^ >> Mg^2+^ for all adsorbents; however, BEI and FeO-BEI showed the highest selectivity for Ba^2+^ among all materials tested. Barium removal from solution was governed by physical adsorption besides ion exchange, intercalation, surface complexation and precipitation, depending mainly on the absorbent type and operational conditions. BEI and FeO-BEI showed a high regenerability (>70–80% desorption efficiency after 5 cycles) and could be considered as efficient sorbent materials for wastewater clean-up.

## 1. Introduction

The accumulation and overconcentration of hazardous metal ions in the aquatic and terrestrial environments of the Earth is a major threat for the ecological system and for human health, as most metal ions are non-biodegradable and tend to cumulate in living organisms, causing diseases and disorders [1,2,3,4]. In the last decades, intense research has been carried out to find advanced and economically attractive solutions, either for the complete elimination of critical metal ions from wastewater or for a concentration reduction below permissible limits set by the local and international standards [5,6]. Currently available wastewater treatment technologies include, for example, chemical precipitation, flocculation, coagulation, electrochemical methods, reverse osmosis, membrane separation and adsorption [7,8]. However, these methods greatly vary in effectiveness and operational costs; thus there is still a high demand for the development of new, efficient, green and low cost adsorbent materials [9,10,11]. Silicate based adsorbents, such as clay minerals and zeolites, can be advantageous over non-siliceous materials or composite materials, because they provide a plenty of adsorption sites due to the very small particle size, high surface area, porous structure and presence of surface functional groups and exchangeable sites, rendering these components ideal for wastewater treatment [12,13,14,15].

Recently, zeolites have received great attention for the remediation of solutions contaminated by critical metal ions (Cd^2+^, Cr^3+^, Cs^+^, Co^2+^, Pb^2+^, Zn^2+^, etc.) and for radioactive waste treatment due to their worldwide abundance, high thermal and radiation stability and uniform three-dimensional pore structure [5]. The short-range-order aluminosilicate phase allophane also exhibits good sorption capacities for certain metal ions, such as Cs^+^, Ba^2+^, Co^2+^, Cu^2+^, Sr^2+^ and Zn^2+^, owning to its unique physicochemical and surface (charge) properties, amphoteric ligand capacity and hollow “nanoball” structure [13,16,17,18]. Smectite group minerals, such as beidellite and montmorillonite, are also in use as adsorbents, ion exchangers and catalysts due to their low cost, high reactivity and worldwide distribution [15,19,20].

Barium (Ba^2+^) can occur in elevated concentrations in different soils, sediments, hydrocarbon deposits, river water and seawater as well as in wastewater of several industries, the military and agriculture [21,22]. The soluble Ba species are highly toxic and, if present in drinking water, may cause severe health problems (e.g., breathing difficulties, increased blood pressure and heart rhythm changes) even in low concentrations [23,24]. High concentrations of Ba^2+^ in drinking water have been associated with partly severe cases of multiple sclerosis and neurodegenerative diseases. Thus, the US Environmental Protection Agency has set the maximum concentration limit for Ba^2+^ in drinking water to 2 mg/L [20]. Apart from that, radioactive Ba (i.e., the isotope ^133^Ba) is one of the most common and most toxic radionuclides present in radioactive liquid waste [25,26]. It has a long half-life and high fission yield [5]. Thus, there is a strong need to separate Ba^2+^ ions from contaminated solutions, but detailed experimental studies on this topic are still scarce.

In this study, the adsorption of aqueous Ba^2+^ by natural and iron oxide modified allophane, beidellite and zeolite was investigated, representing highly promising (efficient, green and low-cost) adsorbents. The effects of contact time, solution pH, temperature, adsorbent dosage, the presence of competing cations, initial Ba^2+^ concentration and the regenerability of the adsorbents were tested. The equilibrium adsorption data were fitted to both the Langmuir and Dubinin–Radushkevich isotherm models. Removal efficiencies and sorption capacities of Ba^2+^ by the novel materials are compared with other available adsorbent materials.

## 2. Materials, Experimental and Methods

### 2.1. Raw Materials

Allophane-rich soil material (ALO) from the allophane facies of the Santo Domingo de los Colorados deposit (Ecuador), bentonite rich in beidellite (BEI) from the “Johanneszeche” talc deposit near Göpfersgrün (Germany) and zeolite (ZEO: white natrolite intergrown with pale pink stilbite) from Pune (India) were used for the adsorption studies, because of their low cost, high abundance and sufficient sorption capacities. ALO and BEI were used as received without further grinding or purification. ZEO was gently crushed in a ball mill for 15 min, following collection of the <32 µm size fraction by wet-sieving to obtain a similar grain size fraction as for the other adsorbents. The total organic carbon content of the raw materials is ≤0.5 wt%; thus the effect of organic matter on the adsorption may be negligible.

### 2.2. Iron(III) Oxide Modification Experiments

**Figure 1:**
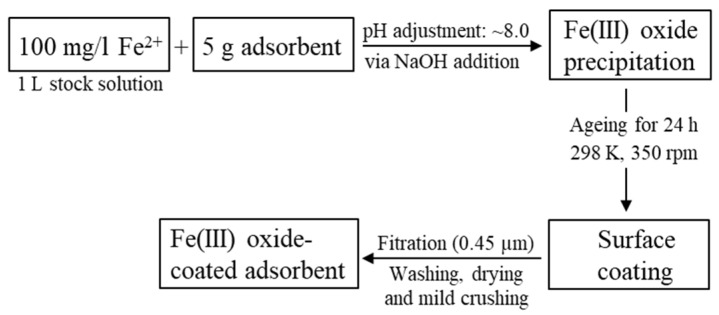
Flow chart showing the preparation of the Fe(III) oxide-coated adsorbents.

Previous investigations have shown that iron(III) oxide-coated materials can exhibit adsorption properties better than the raw materials [27,28]. In order to prepare iron(III) oxide-coated adsorbents (Figure 1), a stock solution (3.0 L) containing 100 mg/L of Fe^2+^ was prepared by the dissolution of iron (II) chloride hydrate (FeCl_2_·H_2_O; ≥98%, from Roth) in ultrapure water (Milli-Q Plus UV, Millipore, Merck, Darmstadt, Germany, 18.2 MΩ at 25 °C). A fraction (5 g) of the raw materials was transferred into 1.0 L of the freshly prepared Fe^2+^ stock solution. The pH of the suspension was adjusted to 8.0 by the addition of droplets of a 0.1 M NaOH solution. This induced the precipitation of iron (III) oxides, such as goethite (FeOOH) and hematite (Fe_2_O_3_). The suspension was aged under oxidizing conditions for 24 h at 298 K. Vigorous stirring of the suspension at 350 rpm ensured a homogenous distribution of the iron (III) oxides at the absorbents surface and a high precipitation quota of the iron (III) oxides. At the end of the experiments, >90% of Fe by weight was removed from the solutions, as determined by measurements of the dissolved Fe concentrations in the stock solution and in the reacted solutions, respectively. The adsorbents were filtered (0.45 μm, Sartorius, cellulose acetate) and washed several times with ultrapure water. The obtained solids were dried at 313 K for 3 days and subsequently gently desegregated using an agate mortar and a pestle. The iron(III) oxide coated adsorbents are hereafter referred to as FeO-ALO, FeO-BEI and FeO-ZEO.

### 2.3. Adsorption Experiments

Stock solutions containing Ba^2+^ in different concentrations were prepared by the dissolution of barium chloride dihydrate (BaCl_2_·2H_2_O, ≥99%, from Roth) in ultrapure water. Batch experiments were conducted at 298 K using a fluid/solid ratio of 500:1 unless otherwise stated.

The effect of contact time on the adsorption of aqueous Ba^2+^ by the (FeO-treated) adsorbents was studied at pH 6.0 ± 0.2 pH units. The kinetic experiments were conducted in 1.5 L high-density polyethylene reactors containing 1 L of the adsorbate (10 mg/L Ba^2+^) and 2.0 g of the adsorbent. Fluid samples (1.5 mL) were taken regularly after 30 s, 2 min, 5 min, 10 min, 30 min, 2 h and 24 h using a syringe (Omnifixᴿ Solo, B.Braun, Maria Enzersdorf, Austria). The liquids were filtered via 0.45 µm cellulose acetate membranes (Sartorius) and acidified using concentrated HNO_3_ of suprapure grade (Roth, ROTIPURANᴿ, Roth, Karlsruhe, Germany).

The effect of pH on the removal efficiency of aqueous Ba^2+^ by (FeO-treated) adsorbents was investigated by reacting 0.4 g of the adsorbent with 200 mL of a solution containing 10 mg/L Ba^2+^. The pH of the suspensions was adjusted to pH 9.0 ± 0.1 with 0.1 M NaOH solutions. The suspensions were stirred at 300 rpm for 1 h to achieve equilibrium conditions. Then, droplets of a 0.05 M HCl solution were added to the suspension to induce a pH decrease from 9.0 to 3.0, each ± 0.1. Fluid samples (1.5 mL) were taken every 0.5–0.7 pH unit steps. These pH drift experiments were of short duration (<2–3 h) to reduce changes in the surface area and charge distribution of the adsorbents.

The effect of the initial Ba^2+^ concentration was studied at pH 6.0 ± 0.2 for 24 h using 50 mL of solution containing Ba^2+^ in different concentrations (5, 10, 25, 50, 100 and 200 mg/L) and 0.1 g of the adsorbent. The experiments were carried out in triplicate at 283 K, 298 K and 313 K in order to study further the effect of temperature on the isotherms.

For the competitive ion tests, local river water (collected from the river Mur, Graz, Austria) was filtered via a 0.45 µm cellulose acetate membrane (Sartorius, Göttingen, Germany). To prevent from the precipitation of Ba-sulfate or Ba-carbonate minerals during the adsorption experiments, the river water was treated as follows: Aqueous SO_4_^2−^ originally present in the river water was removed by the titration with a 0.5 M BaCl_2_ solution to induce barite (BaSO_4_) precipitation. This chemical precipitate was separated by filtration (0.45 μm, Sartorius, cellulose acetate). The solution was then adjusted to a pH of 3.0 by the titration with 0.1 M HCl solutions to induce degassing of CO_2_, thus preventing from the precipitation of witherite (BaCO_3_). Prior to the competitive cation experiments, the pH of the modified river water was readjusted to 6.0 by the addition of 0.1 M NaOH solutions and the aqueous Ba^2+^ concentration set to 40 mg/L by the addition of highly concentrated (3.5 M) BaCl_2_ solutions. Afterwards, 0.1 g of the adsorbent was reacted with 50 mL of the modified river water for 24 h. In an experimental subset, the adsorbent mass was increased to 0.5 g in order to assess the optimum adsorbent dosage.

The regenerability of the adsorbents was evaluated in adsorption–desorption cycles. Therefore, 0.1 g of adsorbent was reacted with 50 mL of a solution containing 10 mg/L Ba^2+^ and let to equilibrate for 0.5 h at pH 6.0. Desorption of Ba^2+^ was induced by pipetting two drops of a 1 M HCl solution to yield a pH of 3.0 ± 0.2, following equilibration of the experiments for 0.5 h, and then readjusting the suspension to pH 6.0 ± 0.1 by the addition of two drops of a 1 M NaOH solution to induce Ba^2+^ adsorption, and waiting for another 0.5 h. Liquid samples (1 mL) were taken after the establishment of adsorption equilibrium at pH 3.0 and 6.0. Five adsorption–desorption cycles were carried out. The regenerability experiments were of relatively short duration (e.g., 5–6 h) to minimize changes in the physicochemical properties of the adsorbents.

The amount of aqueous Ba^2+^ adsorbed by the different (FeO-treated) adsorbents (q_e_ in mg/g) was calculated using Equation (1):

(1) $$q_{e}=\frac{\left(C_{0}-C_{e}\right)}{m}\times V$$

where C_0_ and C_e_ are the initial and the equilibrium concentrations of the adsorbate (in mg/L), m denotes the dry mass of the adsorbent (in g) and V is the volume of the solution (in L).

The efficiency of the adsorption was calculated using Equation (2):

(2) $$\%\mathrm{removal}=\frac{\left(C_{0}-C_{e}\right)}{C_{0}}\times 100$$

where % removal refers to the percentage of removed adsorbate at equilibrium. Note here that the reproducibility of all results was determined in triplicate. Relative standard deviations were found to be always below ±3%. In the following, only the average values are reported.

### 2.4. Analytical Methods

#### 2.4.1. Fluid-Phase Characterization

The pH of the experimental solution was measured at 298 K with a SenTix 41 glass electrode connected to a WTW Multi 350i (Xylem Analytics, Weilheim, Germany), which was calibrated via NIST buffer standard solutions at pH 4.01, 7.00 and 10.00 (analytical error: ±0.05 pH units).

The aqueous concentrations of Al, Ba, Ca, Fe, K, Mg, Na and Si were analyzed in acidified samples (2% HNO_3_ matrix) by inductively coupled plasma optical emission spectroscopy (ICP-OES) using a PerkinElmer Optima 8300 (Waltham, MA, USA). The concentration range considered in the ICP-OES analyses ranged from 1 × 10^−3^ to 5 × 10^2^ mg/L, depending on the analyte type. Detections limits are always below 1 µg/L for the elements of interest. NIST 1640a, in-house and SPS-SW2 Batch 130 standards were measured at the beginning, at the end and in between individual sample sequences [13]. The analytical error is ±2% for Mg and Na and ±3% for Al, Ba, Ca, Fe, K and Si analyses, respectively (2 SD, 3 replicates for each sample).

The aqueous speciation of Ba in solution and the saturation degrees of relevant Ba-bearing minerals, such as Ba-(hydr)oxides, Ba-chloride hydrates and Ba-silicates, were computed with the PHREEQC software and its implemented Lawrence Livermore National Laboratory (LLNL) database at the experimental pH and temperature [29,30].

#### 2.4.2. Solid-Phase Characterization

The mineralogical composition of the (FeO-treated) adsorbents was investigated by powder X-ray diffraction (P-XRD). The materials were prepared in XRD sample holders using the top-loading technique and examined in the range 5–85° 2θ with a step size of 0.008° 2θ and a count time of 40 s per step using a X’Pert PRO diffractometer (PANalytical, Almelo, The Netherlands) operated at 40 kV and 40 mA (Co-Kα) and fitted with a high-speed Scientific X’Celerator detector. Mineral identification and mineral quantification were carried out by Rietveld refinement of the P-XRD patterns using the X’Pert Highscore Plus software (version 2.2e, PANalytical, Almelo, The Netherlands) and the pdf-4 database (analytical uncertainty: ±3 wt%; [31]).

Attenuated total reflectance–Fourier transform infrared spectroscopy (ATR-FTIR) data were acquired on a PerkinElmer Frontier spectrometer (Waltham, MA, USA) for the characterization of the poorly crystallized components present in the (FeO-treated) adsorbents. Mid-infrared (MIR) spectra were recorded in the range from 4000–650 cm^−1^ at a resolution of 2 cm^−1^.

The chemical composition of the raw materials was analyzed by wavelength dispersive X-ray fluorescence (XRF) spectrometry using a PW2404 (PANalytical, Almelo, The Netherlands). Therefore, the loss on ignition (LOI) was determined by gravimetric analysis. Standard glass tablets were produced by fusion in a PANalytical Perl’X 3 bead preparation system (Malvern PANalytical) using 6.0 g of Li_2_B_4_O_7_ as fluent agent. The analytical error is <0.5 wt% for the major elements [32].

The particle shape and the size of the (FeO-treated) adsorbents were characterized by scanning electron microscopy (SEM). The materials were prepared on Al-stubs, coated with Au-Pd and imaged by a DSM 982 Gemini microscope (ZEISS, Oberkochen, Germany) operated at 5 kV of accelerating voltage. Element distribution mappings (Al, Si, Fe, Ba, etc.) were acquired on a Sigma 300 VP (ZEISS, Oberkochen, Germany) operated at 15 kV and equipped with a X-max80 SDD energy dispersive X-ray spectroscopy (EDX, Oxford Instrument, Abingdon, UK) detector. Chemical data were acquired on selected single spots per sample at an analytical precision of <2 at% for the elements of interest (Al, Si, Fe, etc.).

The specific surface area of natural and FeO-modified adsorbents was determined before and after the Ba adsorption by N_2_ physisorption using the single-point adsorption BET method (e.g., [33]) in order to track changes in the surface properties of the adsorbents. All materials were pre-dried as stated above. The measurements were carried out on a Micrometrics FlowSorb II 2300 using a He(69.8)-N_2_(30.2) mixture as the carrier gas at the Institute of Technology and Testing of Construction Materials at Graz University of Technology. The analytical error was determined as ±10%.

## 3. Results and Discussion

### 3.1. Mineralogical Composition of the Adsorbents

**Figure 2:**
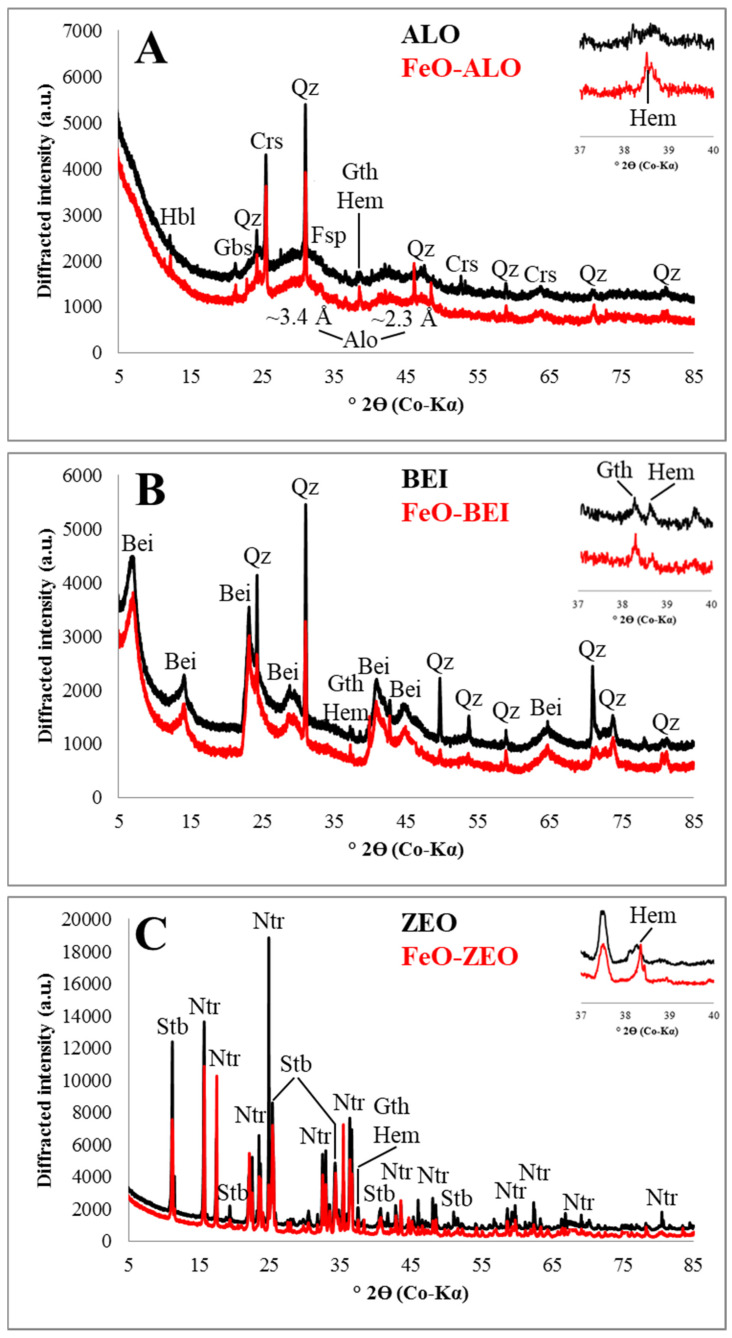
XRD patterns of natural and FeO-modified ALO (**A**), BEI (**B**) and ZEO (**C**). Hbl—hornblende; Gbs—gibbsite; Qz—quartz; Crs—cristobalite; Fsp—feldspar; Alo—allophane; Gth—goethite; Hem—hematite; Bei—beidellite; Stb—stilbite; Ntr—natrolite.

XRD patterns of the adsorbents used in this study are displayed in Figure 2. The mineralogy of the raw (and FeO-modified) materials is reported in Table 1.

ALO consists of allophane (70 wt%), which displays two broad and asymmetric reflections centered at 30.50° 2θ and 45.75° 2θ, which is typical for this short-range-order mineral phase. Minor constituents in ALO are quartz (7 wt%), halloysite (5 wt%), cristobalite, gibbsite, hornblende (each 4 wt%) and goethite (3 wt%). Illite, feldspar (mainly albite-type) and Mg-chlorite (clinochlore) are present as traces (1 wt%). BEI consists of beidellite (91 wt%) as well as quartz (5 wt%) and hematite (4 wt%). The mineralogical composition of ZEO is based on a mixture of natrolite (74 wt%) and stilbite (26 wt%). No significant mineralogical changes of the adsorbents were observed after the FeO-modification, except for the d_101_-peak (24.42° 2θ) and d_301_-peak (38.55° 2θ) of goethite and the d_014_-peak of hematite (38.68° 2θ), which gained intensity (see inserted figures in Figure 2). 

XRD patterns of the adsorbents collected after the adsorption experiments are identical to those of the unreacted materials (see Supplementary Material Figure S1). Importantly, they do not reveal the presence of discrete Ba-bearing minerals due to the low Ba^2+^ concentration relative to the adsorbents mass. This observation may indicate that sorption is the main mechanism governing Ba^2+^ uptake from solution.

**Figure 3:**
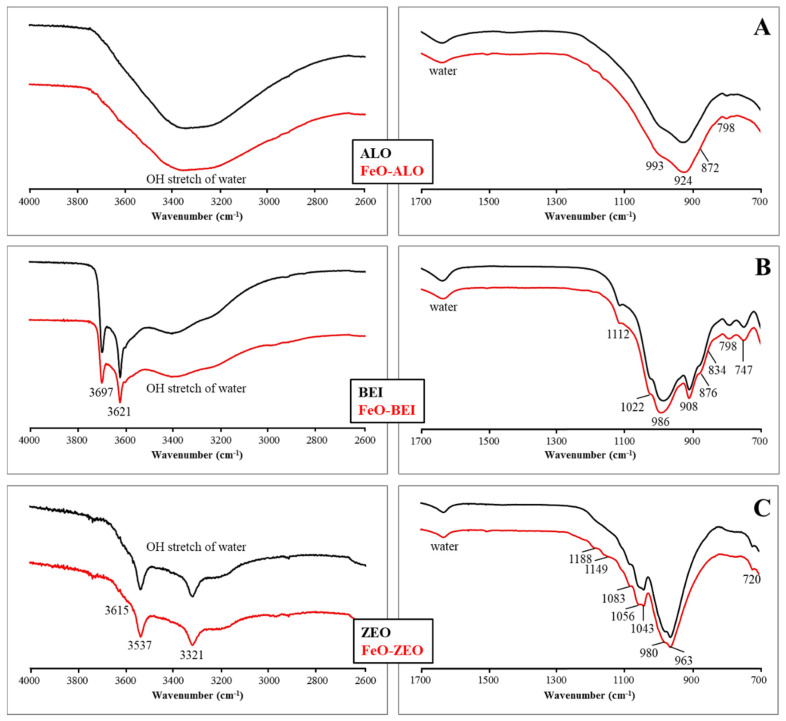
ATR-FTIR spectra of natural and FeO-modified ALO (**A**), BEI (**B**) and ZEO (**C**). Left panel) Hydroxyl stretching region. Right panel) Lattice vibration region.

MIR spectra of the adsorbents with and without FeO-modification are displayed in Figure 3.

ALO and FeO-ALO consist mainly of allophane, as indicated by the strong lattice vibration bands in the region between 1000 and 900 cm^−1^ due to Si–O–(Si) or Si–O–(Al) vibrations and at 870 cm^−1^ due to Si–OH groups [17,34]. Quartz occurs in minor quantities and was identified based on a diagnostic IR band in the lattice vibration region at 798 cm^−1^ [13]. BEI and FeO–BEI display two strong IR bands at 3697 cm^−1^ and 3621 cm^−1^, corresponding to hydroxyl groups associated with octahedral cations in beidellite. The broadness of the 3621 cm^−1^ band documents the substitution of Al^3+^ by Fe^2+^ or Mg^2+^ cations in the octahedral sheet of beidellite, which is further seen by the bending vibrations of hydroxyl groups associated with these octahedral cations at 908 cm^−1^ (Al–Al–OH), 876 cm^−1^ (Al–Fe–OH) and 834 cm^−1^ (Al–Mg–OH) [35]. The IR bands at 1120 cm^−1^, 1022 cm^−1^, 986 cm^−1^ and 798 cm^−1^ correspond to Si–O stretching vibrations in beidellite and quartz, whereas the IR band at 747 cm^−1^ is most likely related to the stretching mode of Al–O–Al linkage [36]. The MIR spectra of ZEO and FeO-ZEO display strong adsorption in the hydroxyl stretching region at 3537 cm^−1^ and 3321 cm^−1^ and a weak one at 3615 cm^−1^, which are attributed to hydroxyl stretching vibrations in zeolitic water in natrolite and stilbite [37]. Numerous and intense IR bands in the lattice vibration region between 1200 and 900 cm^−1^ are due to Si–O–Si and Si–O–Al stretching vibrations. The IR band at 720 cm^−1^ corresponds to the 4-membered rings in the tetrahedral framework of zeolite structures [37]. IR spectra of the materials obtained after the adsorption experiments (Supplementary Material Figure S2) look similar to those of the unreacted adsorbents, except for the ZEO and FeO-ZEO, which displayed minor modifications in the lattice vibration region.

### 3.2. Geochemical and Microstructural Characterization of the Adsorbents

**Figure 4:**
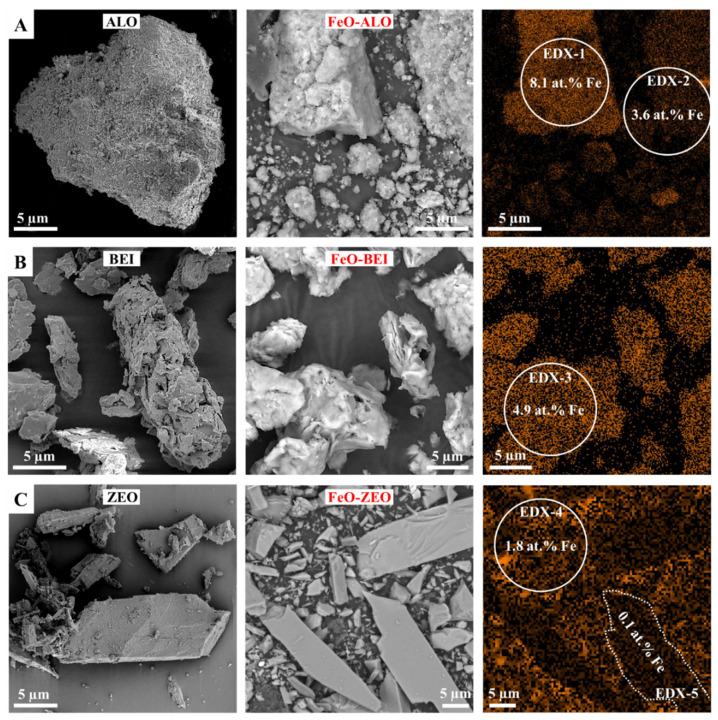
SEM images of natural (first panel) and FeO-modified (second panel) ALO (**A**), BEI (**B**) and ZEO (**C**) adsorbents. Fe distribution maps of the images shown in the second panel are provided on the right. Marked areas indicate positions of EDX measurements (Supplementary Material Figure S3).

The chemical compositions of the adsorbents before and after FeO-modification are reported in Table 2. Representative SEM images of these adsorbents are presented in Figure 4.

ALO and particularly FeO-ALO display µm-sized aggregates of clumped particles rich in allophane having goethite and hematite in different proportions attached onto the allophane surfaces. Individual allophane particles appear as hollow spherule structures. These particles have diameters of 3–5 nm and an atomic Al/Si ratio of 1.3 ± 0.2, according to Baldermann et al. [13]. BEI and FeO-BEI are composed mainly of very fine grained (<1 µm), veil-like to flaky or platy beidellite particles, Ca_0.13_Mg_0.20_Na_0.01_K_0.01_)(Al_1.50_Mg_0.42_Fe^3+^_0.19_)∑_2.11_[Al_0.59_Si_3.41_O_10_](OH)_2_, having goethite and hematite grains attached on the surface, whose concentrations increase after the FeO-modification, as well as a few coarser quartz grains. ZEO and FeO-ZEO are mixtures of needle-shaped to fibrous crystals (1–30 µm in largest dimension) as well as short-prismatic crystals (5–20 µm in diameter) arranged in more compact aggregates. These characteristic particle morphologies can be assigned to natrolite and stilbite, respectively. The Fe distribution map of FeO-ZEO reveals a heterogeneous composition, with the smaller particles (i.e., stilbite) being relatively enriched in Fe and the coarser ones (i.e., natrolite) being depleted in Fe.

The surface areas of the adsorbents increase in the order: ZEO (2.3 m^2^/g) < BEI (50.3 m^2^/g) < ALO (368.5 m^2^/g). No significant change in the surface areas was observed after the FeO-modification (±10% change, which is within the analytical uncertainty of the method) due to the low amounts of Fe added to the adsorbents (2.5% Fe_2_O_3_ by weight). The specific surface areas of all adsorbents measured after the Ba adsorption were found to be identical to those of the unreacted (raw and FeO-coated) materials (5% change), which clearly indicates that changes in the physical properties of the adsorbents during the duration of the experiments are negligible.

### 3.3. Adsorption Studies

#### 3.3.1. Aqueous Speciation of Barium

The affinity between an adsorbent and an adsorbate is an important parameter that controls the extent and rate of an adsorption process [13]. Previous adsorption studies have shown that the aqueous speciation of a given metal ion, solution pH, contact time, temperature, the presence or absence of competitive ions and the metal ion concentration among others are key variables for determining the efficiency, kinetics and the mechanism of an adsorption reaction [7,9,38]. As for this study, these factors control the speed and the magnitude at which aqueous Ba^2+^ gets attached onto ALO, BEI and ZEO with (out) FeO-modification.

In all experiments, the aqueous speciation of Ba was predominated by “free” Ba^2+^ ions (>99%) in the pH range from 9.0 to 3.0 and only less than 2% of the total Ba species belonged to the BaCl^+^ and BaOH^+^ aquo-complexes, demonstrating that (i) the vast majority of Ba^2+^ in solution prevailed in the cationic form and (ii) the aqueous speciation of Ba did not change during the pH-drift experiments. In the case of the competitive ion test, the aqueous speciation of Ba, Ca, K, Mg and Na was dominated by Ba^2+^ (>98%), Ca^2+^ (>99%), K^+^ (100%), Mg^2+^ (>95%) and Na^+^ ions (>98%) over trace amounts of BaCl^+^ (<2%), CaCl^+^ (1%), MgCl^+^ (<5%) and NaCl (<2%) aquo-complexes. All aqueous solutions were at any time undersaturated with respect to the various Ba-bearing mineral phases, such as barite, witherite, barytocalcite (BaCa(CO_3_)_2_), sanbornite (BaSi_2_O_5_), barium hydroxide monohydrate (Ba(OH)_2_·H_2_O) and barium hydroxide octahydrate (Ba(OH)_2_·8H_2_O), corroborating the XRD and FTIR results.

#### 3.3.2. Kinetics Experiments

**Figure 5:**
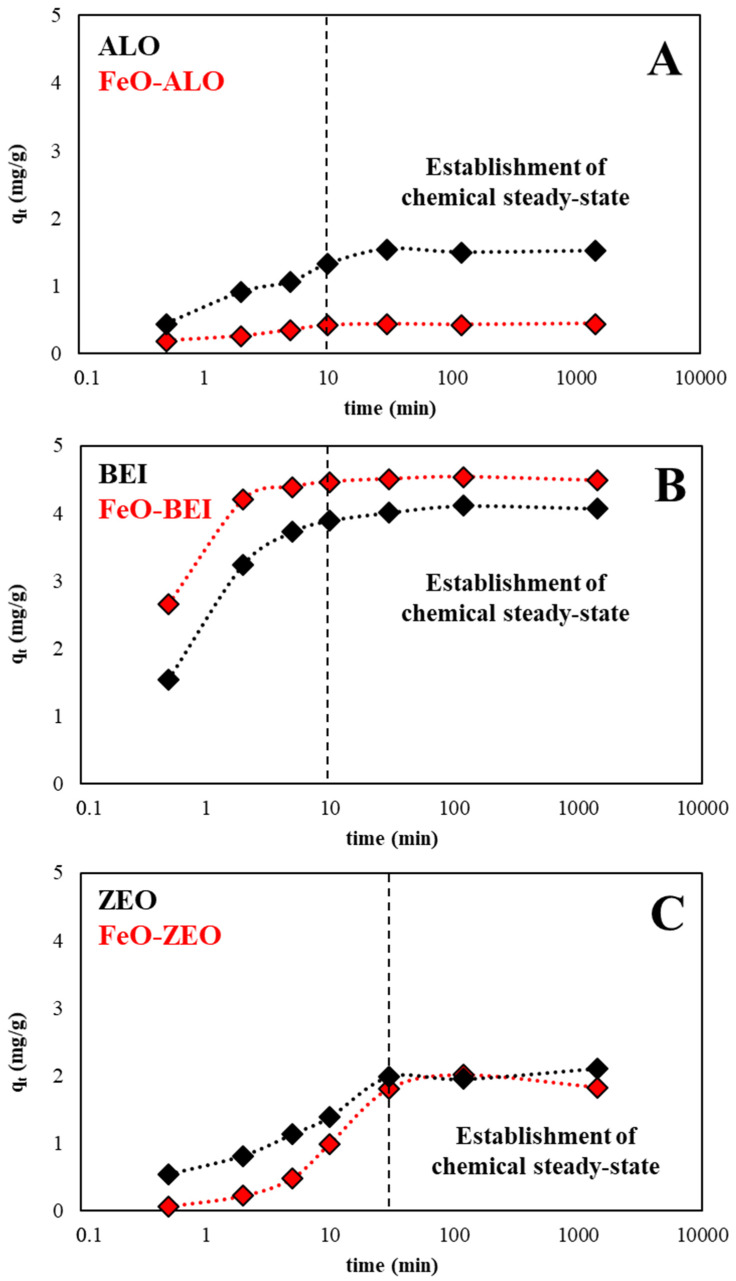
Kinetics experiments (operational conditions: 298 K, pH 6.0, adsorbate/adsorbent ratio: 500:1, 10 mg/L Ba). The effect of contact time on the adsorption of aqueous Ba^2+^ by natural and FeO-modified ALO (**A**), BEI (**B**) and ZEO (**C**) is shown.

The kinetics of the adsorption of aqueous Ba^2+^ by ALO, FeO-ALO, BEI, FeO-BEI, ZEO and FeO-ZEO at pH 6.0 and room temperature are presented in Figure 5. In the first minutes of contact time, the uptake of Ba^2+^ from solution increased rapidly, because of abundant binding sites at the surface of the different adsorbents. However, it slowed down afterwards due to (i) a decrease in the aqueous Ba^2+^ concentration with time, (ii) a lowering of the concentration difference between the bulk solution and the adsorbent–liquid interface and (iii) a decrease of available binding sites at the surface of the adsorbents as the adsorption process continued [39]. Thus, the results of the kinetics experiments indicate that the adsorption of aqueous Ba^2+^ by all adsorbents is a heterogeneous process, with an initial fast adsorption followed by a slower one. The kinetics of the Ba^2+^ adsorption by all adsorbents with(out) FeO-modification were fitted by a pseudo-first-order (PFO) model (Equation (3)):

(3) $$\ln(q_{e}-q_{t})=-k\cdot t+\ln(q_{e})$$

where q_e_ and q_t_ are the amounts of Ba^2+^ taken up from solution per mass of adsorbent (mg/g) at equilibrium and at time t (min) and k (1/min) is the rate constant [40]. Note that the plot of Equation (3) was linear for all adsorbents and that and the calculated and measured q_e_ values matched very well (slope: 0.968; R^2^ = 0.955; *n* = 6), which (i) clearly demonstrated the applicability of the PFO model to fit the experimental data and (ii) revealed that the removal of Ba^2+^ from solution follows physical adsorption. The calculated values for k and q_e_ as well as the removal efficiency and the measured q_e_ values are reported in Table 3. 

It can be concluded that Ba^2+^ removal from solution by all adsorbents was a relatively fast process, which increased in the order: FeO-ZEO and ZEO (30 min) < BEI and ALO (10 min) < FeO-BEI and FeO-ALO (5–10 min). This indicates that BEI and ALO with and without FeO-modification have a higher affinity to adsorb Ba^2+^ ions than ZEO and FeO-ZEO, which is likely related to abundant ≡Si–OH and especially ≡Al–OH groups present in the clayey adsorbents (e.g., [13,41]). FeO-ZEO and ZEO hardly follow pseudo-first-order kinetics, although the calculated R^2^ values are high (>0.96 and 0.99) and the measured and calculated qe values are consistent (Table 3), which suggests that the removal mechanism for Ba^2+^ ions is more complex. It is proposed that initial adsorption corresponded mainly to ion exchange in surface-related micropores, followed by the diffusion of Ba^2+^ into zeolite channels, where they occupied the exchangeable sites (Na^+^, Ca^2+^ and K^+^) within the zeolite crystal structure [39]. 

It can also be seen that the contribution of additional ≡Fe–OH groups, as the result of the FeO-modification, did not significantly increase the adsorption, although such surface sites can attract cations both via electrostatic interaction and surface complexation [42]. However, in view of the removal efficiency of aqueous Ba^2+^ at equilibrium, BEI and FeO-BEI performed about 2-times better than ZEO and FeO-ZEO and about 3-times better than ALO and FeO-ALO. This finding suggests further that the surface area measured for the different adsorbents has only a minor influence on the speed and magnitude of the adsorption process, which corroborates prior conclusions drawn by, e.g., Baldermann et al. [13].

#### 3.3.3. Effect of Solution pH on Ba^2+^ Removal

**Figure 6:**
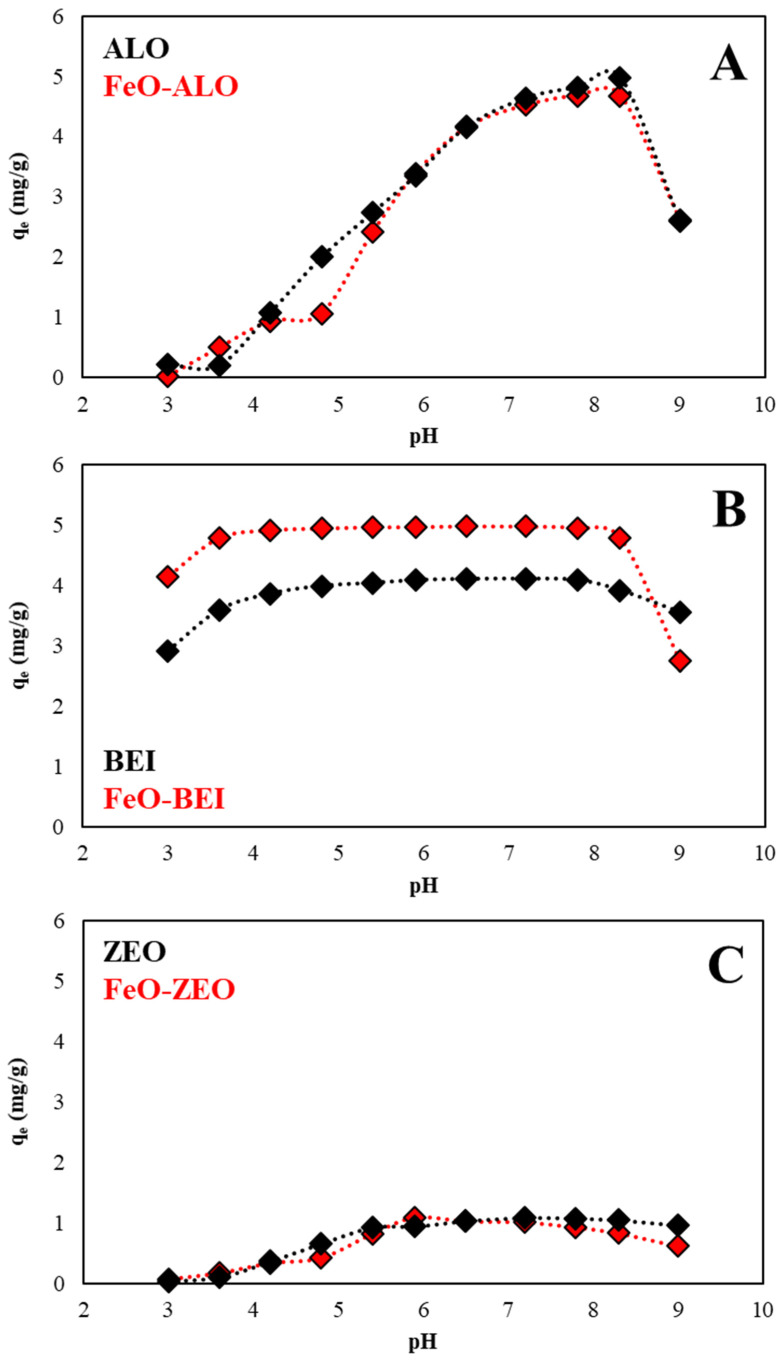
pH-drift experiments (operational conditions: 298 K, adsorbate/adsorbent ratio: 500:1, 10 mg/L Ba). The effect of solution pH on the adsorption of aqueous Ba^2+^ by natural and FeO-modified ALO (**A**), BEI (**B**) and ZEO (**C**) is shown.

The solution pH is of great relevance for an adsorption process, because acidity affects (i) the aqueous speciation of the adsorbate, which is Ba^2+^ in all experiments conducted in this study, (ii) the (de)protonation degree of the surface functional ≡Al–OH and ≡Si–OH groups of the adsorbents [13,41] and (iii) the charge distribution of the adsorbents [11]. The effect of solution pH on the adsorption of aqueous Ba^2+^ by ALO, BEI and ZEO with (out) FeO-modification is seen in Figure 6. 

It is evident that the amount of Ba^2+^ adsorbed from solution increases with increasing pH, because under acidic conditions the ≡Al–OH and ≡Si–OH groups are more protonated, meaning that most of the potential binding sites for Ba^2+^ are occupied by H^+^ ions. As natural zeolites preferentially adsorb H^+^ ions relative to metal ions and antibiotics [39,43,44], Ba^2+^ adsorption by ZEO and FeO-ZEO was low under strongly acidic conditions and increased slightly towards more alkaline conditions, reaching an adsorption optimum at pH 5.5–8.0 (17–22% removal). ALO and FeO-ALO also revealed low adsorption of aqueous Ba^2+^ at pH ≤ 4.0 (≤20% removal) due to repulsive electrostatic forces between the protonated functional groups and Ba^2+^ ions [18,45], but attained a maximum at pH 7.0–8.5 (90–97% removal). This pH-dependency of the adsorption of the ALO-based adsorbents is most likely attributable to the high amount of variable (pH-dependent) charges in natural allophanes [13]. BEI exhibited a good performance in the pH range from 4.0 to 8.5 (80–86% removal). The decrease of Ba^2+^ adsorption at pH ≤ 4.0 can be best explained by the increasing attachment of H^+^ ions onto the surface functional groups in BEI; thus, limiting the number of free binding sites where Ba^2+^ ions can get attached onto. 

FeO-BEI revealed the highest removal efficiency of aqueous Ba^2+^ among all other adsorbents tested in this study (96–99.9% removal at pH 3.5–8.5), which indicates that the surface FeO-coating was stable over a broad pH range, corroborating the results reported previously by Pawar et al. [46]. At pH ≥ 8.5, the increasing abundance of hydroxylated complexes of barium decreased the adsorption capacity for all adsorbents, which is consistent with observations made for other metal ions, such as Cu and Pb [47]. Alternatively, an increased dissolution of traces of reactive siliceous components naturally present in the adsorbents, such as volcanic glass, under alkaline conditions and subsequent formation of Ba-bearing (alumino) silicates onto the surface of the adsorbents could also reduce the efficiency of the adsorption process [13], even though such precipitates were not observed by XRD and FTIR analyses.

#### 3.3.4. Effect of Initial Ba^2+^ Concentration

**Figure 7:**
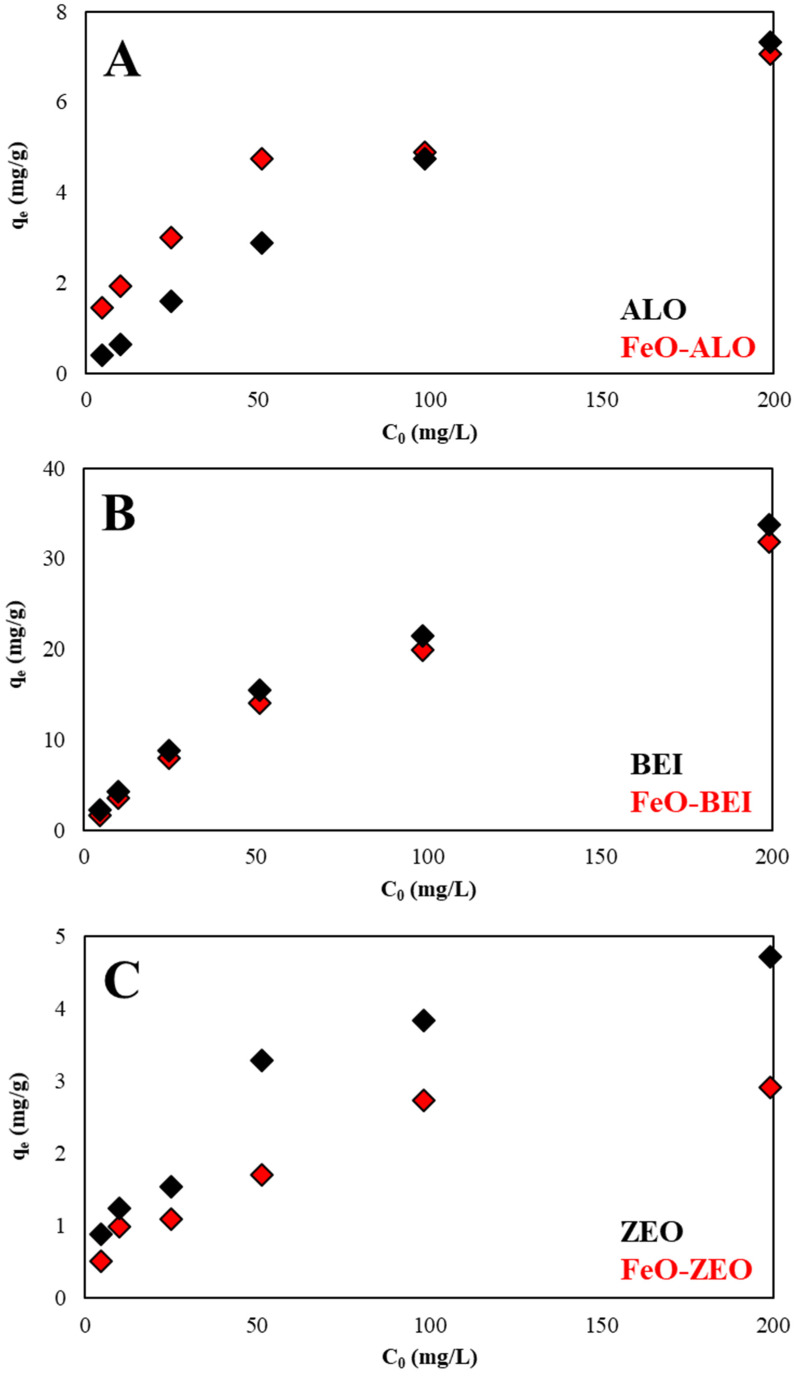
Effect of the initial Ba^2+^ concentration (operational conditions: 298 K, pH 6.0, adsorbate/adsorbent ratio: 500:1, 5–200 mg/L Ba) on the adsorption of aqueous Ba^2+^ by natural and FeO-modified ALO (**A**), BEI (**B**) and ZEO (**C**).

Figure 7 shows the relation between the amount of Ba^2+^ adsorbed by ALO, BEI and ZEO with(out) FeO-modification and the initial concentration of Ba^2+^ in the solution. It is evident that when the Ba^2+^ concentration increased from 5 to 200 mg/L, the q_e_ values increased almost proportionally for all adsorbents. This indicates that the available binding sites became occupied, as the initial Ba^2+^ concentration increased. The FeO-modification had no effect (ALO and BEI) or even a negative one (ZEO) on the removal efficiencies of aqueous Ba^2+^.

#### 3.3.5. Effect of Competing Cations

**Figure 8:**
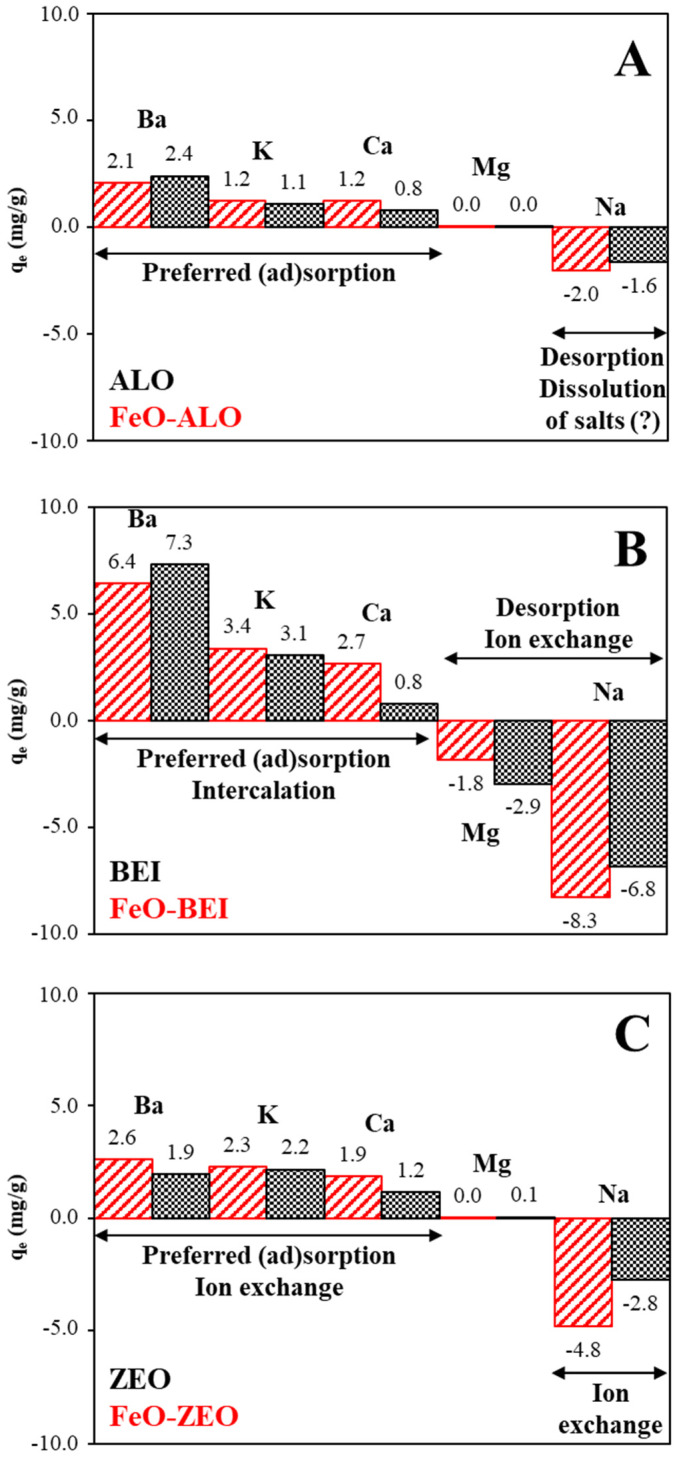
Effect of competing ions (operational conditions: 298 K, pH 6.0, adsorbate/adsorbent ratio: 500:1, cations: 0.85 mmol/L Ca^2+^, 0.20 mmol/L Mg^2+^, 163.2 mol/L Na^+^, 0.38 mmol/L K^+^ and 0.30 mmol/L Ba^2+^) on the adsorption of natural and FeO-modified ALO (**A**), BEI (**B**) and ZEO (**C**).

In practice, waste solutions contain a mixture of different pollutants, such as critical metal ions (which is Ba in this case), as well as harmless solutes of different concentration and type, such as Ca^2+^, Mg^2+^, Na^+^, K^+^, etc. These cations have different affinities to adsorb onto charged surfaces, because of their different ionic properties, such as ion radius, charge, ionic potential, electronegativity, hydration state, hydration enthalpy, etc. [48]. As for this study, local river water contaminated with Ba^2+^ ions was used for the competitive cation tests, which fulfills the requirements mentioned above. Figure 8 shows the adsorption of cations from a multi-component solution.

The multi-component solution originally contained 0.85 mmol/L Ca^2+^, 0.20 mmol/L Mg^2+^, 163.2 mol/L Na^+^ (due to the readjustment of the pH by NaOH), 0.38 mmol/L K^+^ and 0.30 mmol/L Ba^2+^ (due to addition of BaCl_2_ solution). It can be seen that the cation uptake from solution followed in the order: Ba^2+^ > K^+^ > Ca^2+^ >> Mg^2+^ for all adsorbents. The removal efficiency increased in the order: ALO < ZEO < BEI. The FeO-modification neither changed the direction nor the magnitude of the adsorption. The Na^+^ concentration increased relative to the river water, as did the Mg^2+^ concentration in the case of FeO-BEI and BEI, which expressed in negative q_e_ values in Figure 8. This suggests that Na^+^ and also Mg^2+^ ions were released back to the solution upon uptake of the other cations. This behavior indicates that mechanisms other than pure adsorption have to be considered (see Section 3.3.9 for further discussion).

The experimental data indicate further that multiple adsorption cycles are needed to decrease the Ba^2+^ concentration in such multi-component solutions to the guideline limit of 0.7 mg/L (or 0.005 mol/L), as recommended by the WHO for Ba^2+^ in drinking water [49]. However, this result is indeed promising, if considering that the river water contained a great variety of other cations, which could have competed for the adsorption sites. An increase of the adsorbent dosage by 5-times (0.1–0.5 g) resulted in a decrease of the Ba^2+^ concentration to 0.03–0.04 mmol/L for BEI and FeO-BEI, 0.09–0.13 mmol/L for ALO and FeO-ALO and 0.22–0.24 mmol/L for ZEO and FeO-ZEO, respectively. An extrapolation of these data indicates that approximately 0.55–0.60 g of the BEI-based adsorbents are needed to decrease the Ba^2+^ concentration of the river water to below the WHO guideline limit.

#### 3.3.6. Adsorption Isotherms

**Figure 9:**
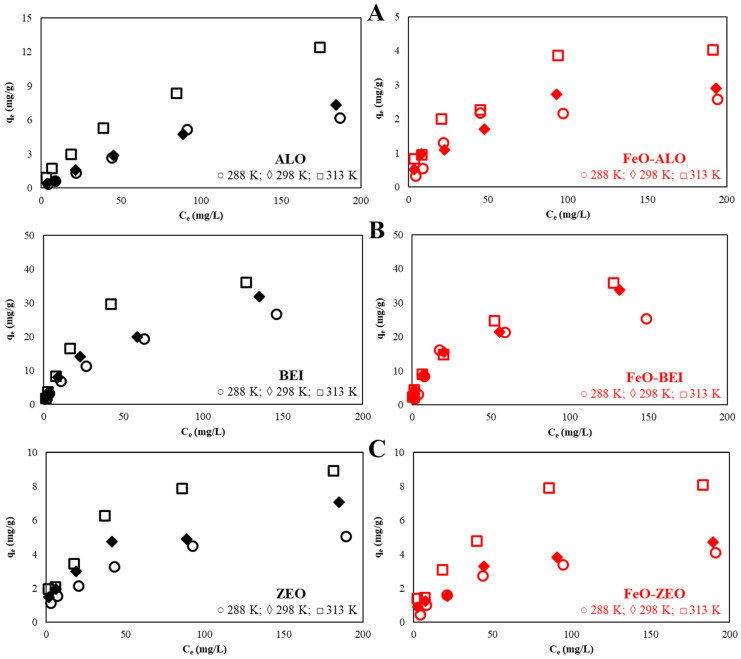
Adsorption isotherms (operational conditions: 283–313 K, pH 6.0, adsorbate/adsorbent ratio: 500:1, 5–200 mg/L Ba) for natural and FeO-modified ALO (**A**), BEI (**B**) and ZEO (**C**). Symbols are larger than the analytical uncertainty.

Adsorption isotherms define the relationship between the concentration of a component in a given aqueous solution and the quantity of the respective component adsorbed onto a solid surface at thermodynamic equilibrium and at constant temperature [46]. The adsorption data obtained in this study (Figure 9) were evaluated using the Langmuir model and the Dubinin–Radushkevich (D–R model) model. These models were chosen for the reasons discussed in detail in Tran et al. [40] and are represented by the Equations (4) and (5) (Langmuir, linear form) and 6–8 (D–R model, linear form), respectively:

(4) $$\frac{C_{e}}{q_{e}}=\frac{1}{K_{L}\cdot Q_{\max}^{0}}+\frac{C_{e}}{Q_{\max}^{0}}$$

(5) $$R_{L}=\frac{1}{1+K_{L}\cdot C_{0}}$$

(6) $$\ln(q_{e})=-K_{\mathrm{DR}}\cdot \varepsilon^{2}+\ln(q_{\mathrm{DR}})$$

(7) $$\varepsilon =R\cdot T\cdot \ln(1+\frac{1}{C_{e}})$$

(8) $$E=\frac{1}{\sqrt{2\cdot K_{\mathrm{DR}}}}$$

where $Q_{\max}^{0}$ and q_DR_ denote the monolayer adsorption capacity of an adsorbent (mg/g), K_L_ (L/mg) and K_DR_ (mol^2^/kJ^2^) are the Langmuir and D–R model adsorption constants, ε is the Polanyi potential (J^2^/mol^2^), R is the universal gas constant (8.314 × 10^−3^ kJ/(mol·K)), T is the temperature (K), R_L_ is the separation factor (i.e., a dimensionless constant that describes the nature of an adsorption process) and E (kJ/mol) is the free energy change during the transfer of one mole of the adsorbate to the surface of the adsorbent. The values for $Q_{\max}^{0}$, K_L_ and R_L_ are reported in Table 4. The values for q_DR_, K_DR_ and E are listed in Table 5, respectively. It can be seen that the experimental data are better described by the Langmuir model than by the D–R model. The values of R_L_ indicate the adsorption process to be favorable for all Ba^2+^ concentrations, temperatures and adsorbents tested in this study, if considering that an adsorption process is irreversible at R_L_ = 0, favorable at 0 < R_L_ < 1, linear at R_L_ = 1 and unfavorable at R_L_ > 1 [40].

An increase in temperature from 288 to 313 K resulted in a higher retention of Ba^2+^ ions. This dependency on temperature may be attributable to an increase of both the active surface sites and pore sizes of the adsorbents [50]. However, after a certain temperature limit is reached, the absorption capacity will start to decrease, which is at app. 318 K or higher for most silicate-based adsorbents [51]. 

The maximum monolayer adsorption capacities are 9.8 mg/g for ZEO, 16.8 mg/g for ALO and 44.8 mg/g for BEI and 9.6 mg/g for FeO-ZEO, 4.6 mg/g for FeO-ALO and 38.6 mg/g for FeO-BEI, respectively, at near-optimum temperature (313 K), which shows that the natural materials were in general advantageous over the FeO-modified ones. The calculated values for E (E < 8 kJ/mol) indicate that the Ba^2+^ uptake from solution follows physical adsorption rather than chemical ion exchange (e.g., [52,53,54]). 

As expected, the adsorption of Ba^2+^ ions depended in particular on the type and on the related physicochemical properties of the natural and FeO-modified adsorbents, as indicated by our data (see Figure 9). This result is in line with the observations prior discussed elsewhere [13,20,55]. Apart from that the removal mechanisms for Ba^2+^ ions from solution by the different adsorbents may be much more complex than indicated by the adsorption isotherms for the reasons discussed in detail in Section 3.3.9.

#### 3.3.7. Thermodynamics

Thermodynamic parameters, such as Gibbs free energy (∆G^0^), enthalpy (∆H^0^) and entropy (∆S^0^), were calculated in order to constrain the feasibility and the nature of the adsorption process (e.g., physical and chemical), according to Equations (9)–(12):

(9) $$\Delta G^{0}=-R\cdot T\cdot {\mathrm{lnK}}_{C}$$

(10) $$\Delta G^{0}=\Delta H^{0}-T\cdot \Delta S^{0}$$

(11) $${\mathrm{lnK}}_{C}=-\frac{\Delta H^{0}}{\mathrm{RT}}\cdot \frac{1}{T}+\frac{\Delta S^{0}}{R}$$

(12) $$K_{C}=K_{L}\cdot 55.5\cdot 1000$$

where K_L_ is the Langmuir constant (see Table 4; multiplied with the molecular weight of Ba (137.33 g/mol) to obtain the unit of L/mmol). K_C_ is the equilibrium constant (dimensionless), which is obtained by the multiplication of K_L_ with the factor of 55.5 (e.g., number of moles of pure water per liter), and then by 1000 [40]. The values for ∆G^0^, ∆H^0^ and ∆S^0^ are reported in Table 6.

The ∆G values suggest that the adsorption of Ba^2+^ by all adsorbent materials with (out) FeO-modification is feasible, spontaneous and more favorable at a higher temperature. The positive values obtained for ∆H indicate the adsorption process to be endothermic. Positive ∆S values suggest an increase in randomness of the adsorption as temperature increases. Similar observations have been made by, e.g., Adeyemo et al. [56] and Uddin [57] and references therein.

#### 3.3.8. Regenerability

**Figure 10:**
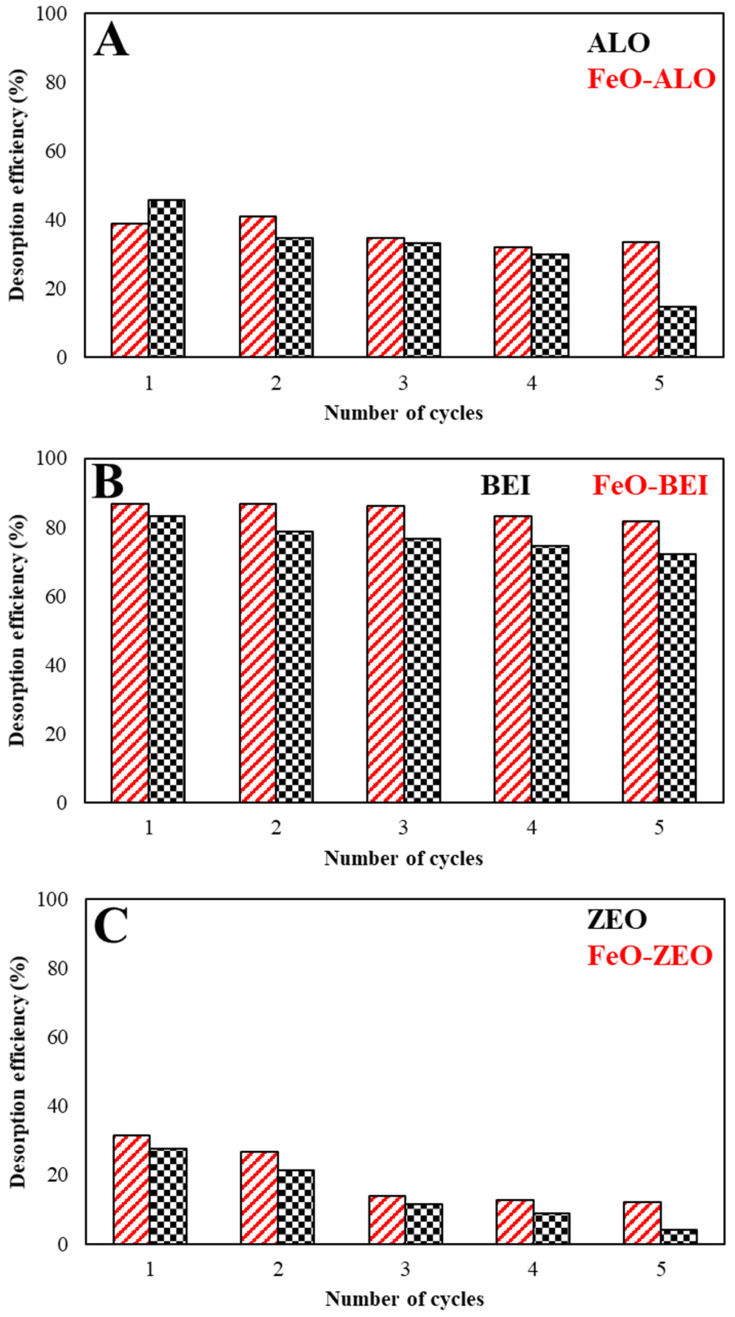
Regenerability (operational conditions: 298 K, pH 6.0–3.0, adsorbate/adsorbent ratio: 500:1, 10 mg/L Ba) of natural and FeO-modified ALO (**A**), BEI (**B**) and ZEO (**C**).

The efficiency, including both sustainability and economic factors, of an adsorption is greatly increased, when (i) the adsorbed pollutant is recoverable via desorption and (ii) the adsorbent is regenerable, i.e., reuse is possible over multiple adsorption–desorption cycles. The amount of Ba^2+^ recovered at the end of each adsorption–desorption cycle (5 in total) is displayed as the desorption efficiency (DE%) in Figure 10 and was calculated with the Equation (13):

(13) $$\mathrm{DE}\;\%=\frac{Mass\;of\;sorbed\;Ba\;\left(mg\right)\;at\;run\;\left(n+1\right)}{Mass\;of\;sorbed\;Ba\;\left(mg\right)\;at\;run\;\left(n\right)}\cdot 100$$

It is evident that the desorption efficiencies of FeO-BEI and BEI remained above 80% and 70% after up to 5 consecutive cycles, which indicates that these adsorbents were neither severely damaged nor chemically modified during the multiple adsorption–desorption cycles, and that their reuse is possible. This is consistent with the mineralogical evaluation of FeO-BEI and BEI obtained after the Ba^2+^ adsorption (Supplementary Material Figures S1 and S2). A similar high regenerability was found for the bentonite-thiourea-formaldehyde composite after Pb^2+^, Mn^7+^ and Cr^6+^ sorption [54]. In contrast, the desorption efficiency of ALO and FeO-ALO was generally lower (40–50%) compared to BEI and FeO-BEI and decreased after 4 cycles, which suggests that the physical and surface charge properties the of these materials are modified under acidic conditions, and that their regenerability is limited. However, mineralogical changes have not been observed for the ALO-based adsorbents upon the regenerability test (Supplementary Material Figures S1 and S2). ZEO and FeO-ZEO displayed by far the lowest regenerability among all adsorbents tested in this study (30%), which decreased dramatically to 5–10% after 5 cycles. FTIR spectra of the both adsorbents revealed intense structural modifications after the multiple adsorption–desorption cycles, making a reuse impossible (Supplementary Material Figure S2).

#### 3.3.9. Uptake Mechanism(s)

Habib and Bockris [58] have stated that the adsorption of an ionic species is unlikely to occur alone, because the developing solid–liquid interface must always be electrically neutral, meaning that there is either ion exchange on the surface or within the pore structure of a certain adsorbent or there might be a counterion in the diffuse layer of a certain adsorbent. Therefore, both the physicochemical properties of the adsorbate (ion radius, charge, ionic potential, electronegativity, hydration state, etc.) and the crystal-chemical parameters of the adsorbent (crystal structure, surface area, cation exchange capacity, etc.) are considered to play a significant role in controlling the direction and the magnitude of a certain adsorption and/or diffusion process. With respect to the adsorbents used in this study, one can conclude (simply speaking) that beidellite is a layered material with an expandable interlayer [15], allophane is an amorphous substance with a hollow “nanoball” structure that is intermitted by defect sites, the so-called perforations [17] and zeolite (natrolite and stilbite) is a crystalline material with a uniform three-dimensional pore structure [5].

**Figure 11:**
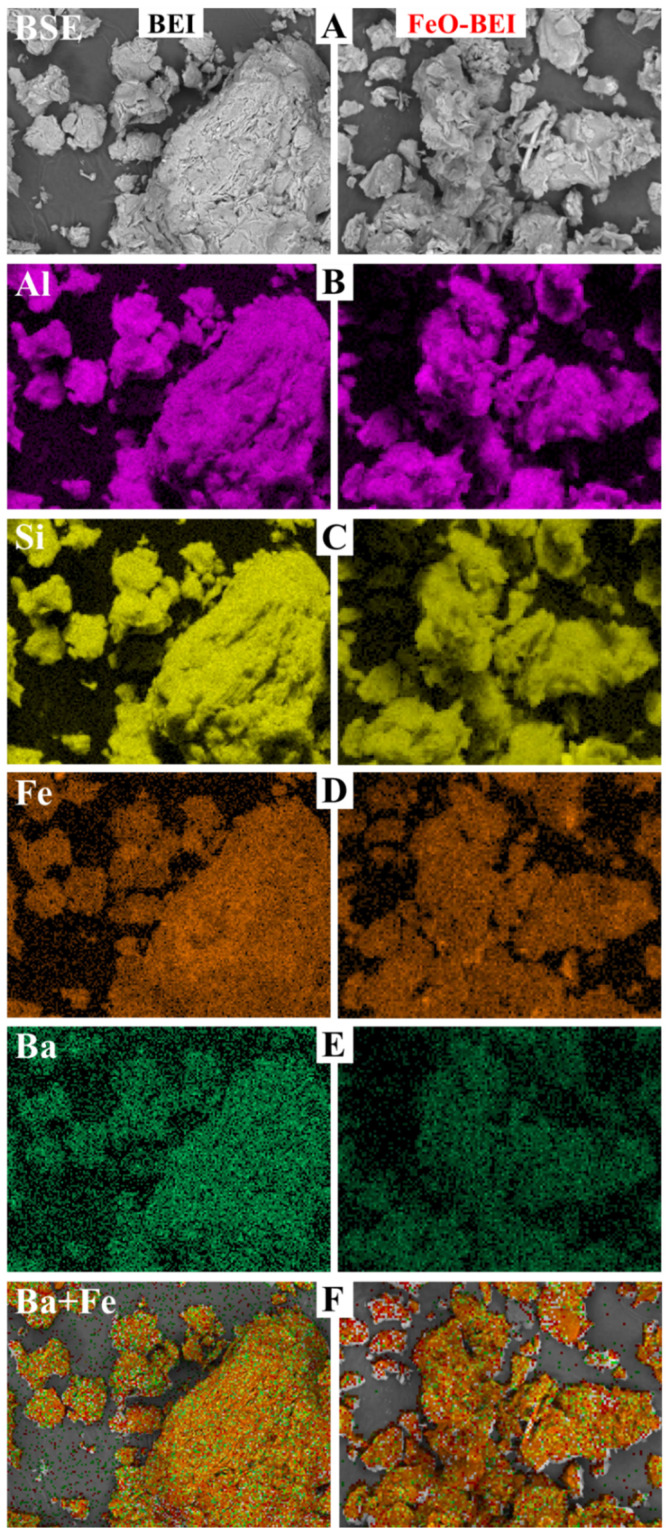
Backscattered electron images (**A**), distribution maps of Al (**B**), Si (**C**), Fe (**D**) and Ba (**E**) and overlay maps (**F**) of Ba (green) + Fe (orange) of BEI and FeO-BEI. Image width: all 15 µm.

The BEI-based adsorbents can expand in water, contrary to the ALO- and ZEO-based materials, making BEI and FeO-BEI more accessible for cations, which explains the high sorption capacities for Ba^2+^ ions (44.8 mg/g and 38.6 mg/g at 313 K). As for the competitive cation test, adsorption–desorption of ionic species on the charged surface as well as intercalation reactions in the interlayer are thought to have caused the release of Na^+^ and Mg^2+^ to the solution and the simultaneous uptake of Ba^2+^, K^+^ and Ca^2+^ ions onto the surface and within the layered structure of BEI and FeO-BEI, balancing charge and keeping electrical neutrality [15,58]. This assertion may be supported by the element distribution maps of BEI and FeO-BEI, shown in Figure 11, which have been collected after the adsorption. The Al and Si distributions belong to octahedral and tetrahedral sites, Fe occupies octahedral sites in beidellite as well as in goethite and hematite due to the FeO-modification, and Ba is the species sorbed onto the surface and/or within the interlayer of beidellite. Note that all elements are homogenously distributed across the sample, which indicates that sorption processes govern mainly the uptake of Ba^2+^ from solution, which is consistent with the interpretation of both the kinetics and adsorption isotherms. These sorption processes are reversible to a high degree, as indicated by the regenerability test (Figure 10).

In the case of ALO and FeO-ALO, it is suggested that Na^+^ ions were liberated from and Ba^2+^ ions were attached onto the defect structural sites and external areas of the allophane nanoball structure. Inner-sphere complexation, formation of molecular clusters of hydrated Ba-complexes within the perforations and real intercalation processes are unlikely to occur, which could be a limiting factor for Ba sorption by allophane-based materials. This interpretation is in line with the conclusions drawn in other adsorption studies [13] and match the results obtained from molecular dynamics calculations of hydrated allophane [59]. 

ZEO is composed of natrolite and stilbite, which are structures with 8 MR (2.6 Å × 3.9 Å) and 9 MR (2.5 Å × 4.1 Å) vs. 8 MR (2.7 Å × 5.6 Å) and 10 MR (4.7 Å × 5.0 Å) pores, respectively [60]. In other words, both zeolite minerals are characterized by small pores, whereby stilbite has a larger porous size than natrolite, but it is present in low quantity (26 wt% vs. 74 wt%) in the adsorbent. The ionic radii of Ba^2+^ and of hydrated Ba^2+^ are about 1.4 Å and 4.1 Å, respectively [61]. Thus, Ba^2+^ can enter inside the surface-related micropores of the zeolite minerals, but it barely fits into the internal pores due to its large hydration sphere, which explains the sluggish kinetics (Figure 5c), the comparable low release of Na^+^ as the counterion (i.e., compensating the negative charge arising from Al of the framework, cf. Figure 8) and the significantly lower sorption capacity of both ZEO and FeO-ZEO compared with the other absorbents (Table 4).

#### 3.3.10. Cost Factor

Activated carbon components are the most frequently used adsorbents of our time. However, their applicability in wastewater treatment is restricted due to a limited regenerability, high costs in the order of US $20–22,000 per ton and increasing difficulties arising from waste production and waste management after their operational use [62]. The results obtained in this study indicate that BEI and subordinate ALO and ZEO with(out) FeO-modification have a high affinity to bind Ba^2+^ ions over a wide pH range. Estimated mining costs for ALO are US $200–600 per ton, which is higher than other economic smectite-clay deposits, such as the Wyoming bentonite and beidellite clays (US $30–300 per ton), but significantly lower than low- to high purify zeolites (US $300–20,000 per ton; [13]). The FeO-modification process, if required to increase the regenerability of a certain adsorbent, may increase the price per ton by <10–20%. The application of alternating layers of natural and FeO-modified BEI and other low-cost adsorbents in filter systems for wastewater treatment could be considered for a better cost-effectiveness.

### 3.4. Comparison of Adsorbents

Table 7 shows a comparison between the adsorption capacities for aqueous Ba^2+^ obtained in this study and those reported in previous studies using similar and other adsorbents. It can be seen that ALO, BEI and ZEO with and without FeO-modification are comparable to most other adsorbents, although such an evaluation may be erroneous for the reasons discussed in De Gisi et al. [62].

The materials tested in this study were not among the highest available, such as Ti_3_C_2_T_x_ Mxene [50], functionalized polymer (SASB-type) nanoparticles [70], phillipsite-chabazite-rich tuff [25] and synthetic zeolites [65]. However, comparably high uptake capacities for aqueous Ba^2+^ by BEI and FeO-BEI were obtained, compared to, for example, dolomite [24], expended perlite [68], (hydrous) metal oxides [66,67], other clay minerals [19,20], nanoscale zero valent iron [42] and pecan shell-based activated carbon [71]. 

Indeed, BEI and, to a lesser extent, FeO-BEI exhibited a superior efficiency to adsorb Ba^2+^ ions over a broad pH range (pH 4.5–8.5) and both materials showed a high regenerability (70–80%) even after five adsorption–desorption cycles. Furthermore, the expected low price and the worldwide occurrence make especially BEI an interesting candidate material for Ba^2+^ removal from solution; yet, its performance in large-scale applications has to be proven.

## 4. Conclusions

The adsorption behavior of aqueous Ba^2+^ by natural and FeO-modified allophane (ALO and FeO-ALO), beidellite (BEI and FeO-BEI) and zeolite (ZEO and FeO-ZEO) was analyzed in kinetic and equilibrium experiments as a function of different operating parameters, such as contact time, pH, initial Ba^2+^ concentration, temperature, presence of competing cations and adsorbent dosage. The results obtained from the kinetic studies indicate that the adsorption of Ba^2+^ was completed within 5–30 min and followed the pseudo-first-order reaction model. Ideal pH and temperature conditions for Ba^2+^ removal from solution were pH 7.5–8.0 and 313 K for all adsorbents. The adsorption data fitted to the Langmuir isotherm model, yielding maximum adsorption capacities for BEI (44.8 mg/g) and FeO-BEI (38.6 mg/g), relative to the other ALO- and ZEO-based adsorbents (3.0–16.8 mg/g). The adsorption process is mainly based on physical adsorption and follows in the order: Ba^2+^ > K^+^ > Ca^2+^ >> Mg^2+^ for all adsorbents. The results of this study show that BEI and FeO-BEI are useful adsorbents for the removal of Ba^2+^ from aqueous solution.

## Figures and Tables

**Table 1:** Mineralogical composition of ALO, BEI and ZEO used as adsorbents.

Phase (wt%)	ALO	BEI	ZEO
Allophane	70	–	–
Beidellite	–	91	–
Clinochlore	1	–	–
Cristobalite	4	–	–
Feldspar	1	–	–
Quartz	7	5	–
Gibbsite	4	–	–
Goethite	3	–	–
Halloysite	5	–	–
Hematite	–	4	–
Hornblende	4	–	–
Illite	1	–	–
Natrolite	–	–	74
Stilbite	–	–	26
SUM	100	100	100

**Table 2:** Chemical composition of ALO, BEI and ZEO. During the FeO-modification about 2.5 wt% of Fe_2_O_3_ was added to each raw material (FeO-ALO, FeO-BEI and FeO-ZEO).

Comp. (wt%)	ALO	BEI	ZEO
LOI	31.7	15.3	13.6
Na_2_O	0.2	0.1	10.8
MgO	0.8	5.2	–
Al_2_O_3_	27.4	22.2	23.2
SiO_2_	31.1	47.7	49.0
P_2_O_5_	0.0	0.6	–
K_2_O	0.1	0.1	0.2
CaO	0.4	1.5	3.1
TiO_2_	0.9	0.2	–
MnO	0.1	–	–
Fe_2_O_3_	7.3	7.2	–
SrO	–	–	0.3
SUM	100.0	100.0	99.9

**Table 3:** Summary of the kinetics experiments. Reported are the equilibration time, the removal efficiencies, the measured (q_e_^†^) and calculated (q_e_^‡^) adsorption capacities and the kinetic parameters for the adsorption of Ba^2+^ by ALO, BEI and ZEO with(out) FeO-modification.

				Pseudo-First Order Parameters		
Adsorbent	Equil. Time	q_e_^†^	Removal Efficiency	k	q_e_^‡^	R^2^
Material	(min)	(mg/g)	(%Removal)	(1/min)	(mg/g)	(-)
ALO	10	1.5	30.5	0.185	1.2	0.940
FeO-ALO	5	0.4	8.8	0.308	0.8	0.981
BEI	10	4.0	80.6	0.787	3.6	0.983
FeO-BEI	5	4.5	89.6	1.266	3.6	0.970
ZEO	30	2.0	40.3	0.060	1.8	0.963
FeO-ZEO	30	1.9	37.6	0.065	2.1	0.999

**Table 4:** Summary of the Langmuir isotherm parameters and separation factors (R_L_) for the adsorption of aqueous Ba^2+^ by ALO, BEI and ZEO with (out) FeO-modification.

			Langmuir Parameters		
Adsorbent	Temperature	Q^0^_max_	K_L_	R^2^	R_L_
Material	(K)	(mg/g)	(L/mg)	(-)	(-)
ALO	288	11.4	0.006	0.935	0.42–0.97
ALO	298	13.2	0.007	0.944	0.42–0.97
ALO	313	16.8	0.014	0.945	0.26–0.93
FeO-ALO	288	3.0	0.031	0.987	0.14–0.86
FeO-ALO	298	3.4	0.032	0.969	0.14–0.87
FeO-ALO	313	4.6	0.036	0.977	0.12–0.85
BEI	288	33.1	0.025	0.984	0.17–0.89
BEI	298	38.0	0.029	0.941	0.15–0.88
BEI	313	44.8	0.034	0.993	0.13–0.85
FeO-BEI	288	29.4	0.044	0.989	0.10–0.82
FeO-BEI	298	36.0	0.055	0.963	0.08–0.79
FeO-BEI	313	38.6	0.058	0.945	0.19–0.90
ZEO	288	5.6	0.044	0.988	0.10–0.81
ZEO	298	7.5	0.045	0.960	0.10–0.83
ZEO	313	9.8	0.051	0.983	0.09–0.80
FeO-ZEO	288	4.9	0.026	0.996	0.16–0.88
FeO-ZEO	298	5.4	0.032	0.966	0.14–0.87
FeO-ZEO	313	9.6	0.033	0.972	0.29–0.94

**Table 5:** Summary of the Dubinin–Radushkevich parameters and mean free energy changes (E) of the adsorption of aqueous Ba^2+^ by ALO, BEI and ZEO with (out) FeO-modification.

			Dubinin-Radushkevich Parameters		
Adsorbent	Temperature	q_DR_	K_DR_	R^2^	E
Material	(K)	(mg/g)	(mol^2^/kJ^2^)	(-)	(kJ/mol)
ALO	288	5.1	3.94E-08	0.943	3.6
ALO	298	5.3	3.63E-08	0.923	3.7
ALO	313	9.2	2.77E-08	0.934	4.3
FeO-ALO	288	2.7	2.97E-08	0.983	4.1
FeO-ALO	298	2.5	2.00E-08	0.892	5.0
FeO-ALO	313	3.5	1.97E-08	0.891	5.0
BEI	288	22.2	2.31E-08	0.974	4.70
BEI	298	25.8	2.11E-08	0.979	4.9
BEI	313	35.7	2.29E-08	0.988	4.7
FeO-BEI	288	27.6	2.37E-08	0.980	4.6
FeO-BEI	298	22.9	1.09E-08	0.923	6.8
FeO-BEI	313	22.8	9.06E-09	0.878	7.4
ZEO	288	4.3	1.58E-08	0.902	5.6
ZEO	298	5.4	1.27E-08	0.871	6.3
ZEO	313	6.8	1.07E-08	0.729	6.8
FeO-ZEO	288	3.9	2.83E-08	0.985	4.2
FeO-ZEO	298	3.8	1.81E-08	0.850	5.3
FeO-ZEO	313	6.6	1.81E-08	0.815	5.3

**Table 6:** Summary of thermodynamic parameters: Gibbs free energy, enthalpy and entropy for the adsorption of aqueous Ba^2+^ by ALO, BEI and ZEO with(out) FeO-modification.

		∆G^0^ (kJ/mol)		∆H^0^ (kJ/mol)	∆S^0^ (kJ/mol·K)
Adsorbent	288 K	298 K	313 K		
ALO	−25.69	−26.97	−30.13	3.13	0.022
FeO-ALO	−29.63	−30.73	−32.59	0.54	0.014
BEI	−29.11	−30.49	−32.44	1.10	0.016
FeO-BEI	−30.46	−32.07	−33.83	0.96	0.016
ZEO	−30.46	−31.60	−33.49	0.54	0.015
FeO-ZEO	−29.20	−30.74	−32.36	0.82	0.015

**Table 7:** Comparison of the adsorption capacities of various materials for the adsorption of Ba^2+^ from solution under different operational conditions. TS—this study.

	Q^0^_max_	Surface Area/	Conc. Range	Equil. Time	T	(Optimum)	
Adsorbent	(mg/g)	Particle Size	of Pollutant	(h)	(°C)	PH	Reference
Alk-Ti_3_C_2_T_x_	46.5	76.4 m^2^/g	50–500 mg/L	24	RT	7.0–10.0	[63]
Aloe Vera biosorbent	107.5	<125 µm	5–200 mg/L	1	20	5.0	[64]
Ash (thermal power plant)	15.1	7 m^2^/g	50–1000 mg/L	0.5	50	4.0–5.0	[65]
Ca-clinoptilolite	15.3	<75 µm	0.005–0.1 N	168	RT	–	[20]
Ca–montmorillonite	15.3	<75 µm	0.005–0.1 N	48	RT	–	[20]
Chromium(IV) oxide	0.1	100–150 µm	0.01–1370 mg/L	1.5	60	11.4	[66]
Dolomite	4.0	20 µm	10–50 mg/L	2	20	5.5	[24]
Hydrous ceric oxide	0.1	120 µm	0.01–1370 mg/L	1	60	11.4	[67]
Expanded perlite	2.5	20 µm	5–50 mg/L	1.5	20	6.0	[68]
Kaolinite	14.8	<38 µm	0.002–214 mg/L	288	RT	>8.0	[19]
Mixed chlorite-illite	16.7	<38 µm	0.002–214 mg/L	144	RT	>8.0	[19]
MOF-based Ba traps	131.1	0.4 m^2^/g	10–1000 mg/L	0.1	25	5.8	[26]
Montmorillonite	20.8	<38 µm	0.002–214 mg/L	192	RT	>8.0	[19]
Ti_3_C_2_T_x_ Mxene	175.1	10 m^2^/g	0.2–10 g/L	1	40	7.0–9.0	[50]
Natural allophane	10.6	294 m^2^/g	1–100 mg/L	0.2	25	8.5	[13]
Natural clinoptilolite	41.1	224–500 µm	0.1 N	72	25	–	[69]
Na-clinoptilolite	109.6	224–500 µm	0.1 N	73	26	–	[69]
Nano-polymer SAB	57.7	4.3 m^2^/g	10–500 mg/L	8	20	8.0	[70]
Nano-polymer SASB	210.4	5.9 m^2^/g	10–500 mg/L	6	20	8.0	[70]
Nanoscale zero valent iron	22.6	20–100 nm	0.1–137 mg/L	4	25	–	[42]
Pecan shell act. carbon	3.3	0.4 m^2^/g	25–100 mg/L	0.5	70	6.0–10.0	[71]
Synthetic allophane-1	38.6	358 m^2^/g	1–100 mg/L	0.2	25	8.5	[13]
Synthetic allophane-2	17.2	370 m^2^/g	1–100 mg/L	0.2	25	8.5	[13]
Ti_3_C_2_T_x_	12.0	9.8 m^2^/g	50–500 mg/L	24	RT	7.0–10.0	[63]
Zeolite-rich tuff	230.2	0.3–0.6 mm	50–500 mg/L	0.5	22	7.7	[25]
Zeolite Z70-4	17.0	25 m^2^/g	50–1000 mg/L	0.5	50	4.0–5.0	[65]
Zeolite Z90-4	119.0	40 m^2^/g	50–1000 mg/L	0.5	50	4.0–5.0	[65]
Zeolite Z90-15	117.7	122 m^2^/g	50–1000 mg/L	0.5	50	4.0–5.0	[65]
ALO	16.8	368.5 m^2^/g	5–200 mg/L	0.2	40	7.5–8.5	TS
FeO-ALO	4.6	342.1 m^2^/g	5–200 mg/L	0.1	40	7.5–8.5	TS
BEI	44.8	50.3 m^2^/g	5–200 mg/L	0.2	40	7.5–8.5	TS
FeO-BEI	38.6	50.1 m^2^/g	5–200 mg/L	0.1	40	7.5–8.5	TS
ZEO	9.8	2.3 m^2^/g	5–200 mg/L	0.5	40	7.5–8.5	TS
FeO-ZEO	9.6	2.6 m^2^/g	5–200 mg/L	0.5	40	7.5–8.5	TS

## Supplementary Materials

The following are available online at https://www.mdpi.com/1996-1944/13/11/2582/s1, Figure S1: XRD patterns of adsorbents collected after the Ba adsorption, Figure S2: FTIR spectra of adsorbents collected after the Ba adsorption, Figure S3: EDX spectra obtained from single spot analyses (areas are marked in Figure 4).

## References

[1] Bacquart T., Frisbie S., Mitchell E., Grigg L., Cole C., Small C., Sarkar B. (2015). Multiple inorganic toxic substances contaminating the groundwater of Myingyan Township, Myanmar: Arsenic, manganese, fluoride, iron, and uranium. Sci. Total. Environ..

[2] Malik Q.A., Khan M.S. (2016). Effect on Human Health due to Drinking Water Contaminated with Heavy Metals. J. Pollut. Eff. Cont..

[3] Chabukdhara M., Gupta S.K., Kotecha Y., Nema A.K. (2017). Groundwater quality in Ghaziabad district, Uttar Pradesh, India: Multivariate and health risk assessment. Chemosphere.

[4] Baldermann A., Landler A., Mittermayr F., Letofsky-Papst I., Steindl F., Galan I., Dietzel M. (2019). Removal of heavy metals (Co, Cr, and Zn) during calcium–aluminium–silicate–hydrate and trioctahedral smectite formation. J. Mater. Sci..

[5] Araissi M., Ayed I., Elaloui E., Moussaoui Y. (2016). Removal of barium and strontium from aqueous solution using zeolite 4A. Water Sci. Technol..

[6] Dhingra N., Singh N.S., Parween T., Sharma R., Oves M., Ansari M., Zain Khan M., Shahadat M., M.I. Ismail I. (2020). Heavy Metal Remediation by Natural Adsorbents. Modern Age Waste Water Problems.

[7] Fu F., Wang Q. (2011). Removal of heavy metal ions from wastewaters: A review. J. Environ. Manag..

[8] Carolin C.F., Kumar P.S., Saravanan A., Joshiba G.J., Naushad M. (2017). Efficient techniques for the removal of toxic heavy metals from aquatic environment: A review. J. Environ. Chem. Eng..

[9] Babel S., Kurniawan T.A. (2003). Low-cost adsorbents for heavy metals uptake from contaminated water: A review. J. Hazard. Mater..

[10] Mukai H., Hirose A., Motai S., Kikuchi R., Tanoi K., Nakanishi T.M., Yaita T., Kogure T. (2016). Cesium adsorption/desorption behavior of clay minerals considering actual contamination conditions in Fukushima. Sci. Rep..

[11] Mukhopadhyay R., Bhaduri D., Sarkar B., Rusmin R., Hou D., Khanam R., Sarkar S., Biswas J.K., Vithanage M., Bhatnagar A. (2020). Clay-polymer nanocomposites: Progress and challenges for use in sustainable water treatment. J. Hazard. Mater..

[12] Baldermann A., Warr L.N., Letofsky-Papst I., Mavromatis V. (2015). Substantial iron sequestration during green-clay authigenesis in modern deep-sea sediments. Nat. Geosci..

[13] Baldermann A., Grießbacher A.C., Baldermann C., Purgstaller B., Letofsky-Papst I., Kaufhold S., Dietzel M. (2018). Removal of barium, cobalt, strontium, and zinc from solution by natural and synthetic allophane adsorbents. Geosciences.

[14] Abu-Danso E., Peräniemi S., Leiviskä T., Kim T.Y., Tripathi K.M., Bhatnagar A. (2020). Synthesis of clay-cellulose biocomposite for the removal of toxic metal ions from aqueous medium. J. Hazard. Mater..

[15] Kakaei S., Khameneh E.S., Rezazadeh F., Hosseini M.H. (2020). Heavy metal removing by modified bentonite and study of catalytic activity. J. Mol. Struct..

[16] Clark C.J., McBride M.B. (1984). Chemisorption of Cu(II) and Co(II) on allophane and imogolite. Clays Clay Miner..

[17] Parfitt R.L. (1990). Allophane in New Zealand—A review. Aust. J. Soil Res..

[18] Wu Y., Lee C.-P., Mimura H., Zhang X., Wei Y. (2018). Stable solidification of silica-based ammonium molybdophosphate by allophane: Application to treatment of radioactive cesium in secondary solid wastes generated from fukushima. J. Hazard. Mater..

[19] Eylem C., Erten H.N., Görktürk H. (1990). Sorption-Desorption Behaviour of Barium on Clays. J. Environ. Radioactivity.

[20] Chávez M.L., de Pablo L., García T.A. (2010). Adsorption of Ba^2+^ by Ca-exchange clinoptilolite tuff and montmorillonite clay. J. Hazard. Mater..

[21] Purdey M. (2004). Chronic barium intoxication disrupts sulphated proteoglycan synthesis: A hypothesis for the origins of multiple sclerosis. Med. Hypotheses.

[22] Charbonnier Q., Moynier F., Bouchez J. (2018). Barium isotope cosmochemistry and geochemistry. Sci. Bull..

[23] Baeza-Alvarado M.D., Olguín M.T. (2011). Surfactant-modified clinoptilolite-rich tuff to remove barium (Ba^2+^) and fulvic acid from mono- and bi-component aqueous media. Micropor. Mesopor. Mat..

[24] Ghaemi A., Torab-Mostaedi M., Ghannadi-Maragheh M. (2011). Characterizations of strontium(II) and barium(II) adsorption from aqueous solutions using dolomite powder. J. Hazard. Mater..

[25] Pepe F., de Gennaro B., Aprea P., Caputo D. (2013). Natural zeolites for heavy metals removal from aqueous solutions: Modeling of the fixed bed Ba^2+^/Na^+^ ion-exchange process using a mixed phillipsite/chabazite-rich tuff. Chem. Eng. J..

[26] Peng Y., Huang H., Liu D., Zhong C. (2016). Radioactive Barium Ion Trap Based on Metal-Organic Framework for Efficient and Irreversible Removal of Barium from Nuclear Wastewater. ACS Appl. Mater. Interfaces.

[27] Nguyen T.C., Loganathan P., Nguyen T.V., Vigneswaran S., Kandasamy J., Naidu R. (2015). Simultaneous adsorption of Cd, Cr, Cu, Pb, and Zn by an iron-coated Australian zeolite in batch and fixed-bed column studies. Chem. Eng. J..

[28] Baragaňo D., Alonso J., Gallego J.R., Lobo M.C., Gil-Díaz M. (2020). Zero valent iron and goethite nanoparticles as new promising remediation techniques for As-polluted soils. Chemosphere.

[29] Parkhurst D.L., Appelo C.A.J. (2013). Description of input and examples for PHREEQC version 3-A computer program for speciation, batch-reaction, one-dimensional transport, and inverse geochemical calculations. U.S. Geol. Surv. Tech. Methods.

[30] Voigt M., Pearce C.R., Baldermann A., Oelkers E.H. (2018). Stable and radiogenic strontium isotope fractionation during hydrothermal seawater-basalt interaction. Geochim. Cosmochim. Acta.

[31] Baldermann A., Abdullayev E., Taghiyeva Y., Alasgarov A., Javad-Zada Z. (2020). Sediment petrography, mineralogy and geochemistry of the Miocene Islam Dağ Section (Eastern Azerbaijan): Implications for the evolution of sediment provenance, palaeo-environment and (post-)depositional alteration patterns. Sedimentology.

[32] Richoz S., Baldermann A., Frauwallner A., Harzhauser M., Daxner-Höck G., Klammer D., Piller W.E. (2017). Geochemistry and mineralogy of the Oligo-Miocene sediments of the Valley of Lakes, Mongolia. Palaeobio. Palaeoenv..

[33] Mavromatis V., Immenhauser A., Buhl D., Purgstaller B., Baldermann A., Dietzel M. (2017). Effect of organic ligands on Mg partitioning and Mg isotope fractionation during low-temperature precipitation of calcite in the absence of growth rate effects. Geochim. Cosmochim. Acta..

[34] Levard C., Doelsch E., Basile-Doelsch I., Abidin Z., Miche H., Masion A., Rose J., Borschneck D., Bottero J.-Y. (2012). Structure and distribution of allophanes, imogolite and proto-imogolite in volcanic soils. Geoderma.

[35] Baldermann A., Dohrmann R., Kaufhold S., Nickel C., Letofsky-Papst I., Dietzel M. (2014). The Fe-Mg-saponite solid solution series—A hydrothermal synthesis study. Clay Miner..

[36] Roy J., Bandyopadhyay N., Das S., Maitra S. (2011). Studies on the Formation of Mullite from Diphasic Al_2_O_3_-SiO_2_ Gel by Fourier Transform Infrared Spectroscopy. Iran. J. Chem. Chem. Eng..

[37] Ma Y., Liu Z., Geng A., Vogt T., Lee Y. (2016). Structural and spectroscopic studies of alkali-metal exchanged stilbites. Micropor. Mesopor. Mat..

[38] Burakov A.E., Galunin E.V., Burakova I.V., Kucherova A.E., Agarwal S., Tkachev A.G., Gupta V.K. (2018). Adsorption of heavy metals on conventional and nanostructured materials for wastewater treatment purposes: A review. Ecotoxicol. Environ. Saf..

[39] Motsi T., Rowson N.A., Simmons M.J.H. (2009). Adsorption of heavy metals from acid mine drainage by natural zeolite. Int. J. Miner. Process..

[40] Tran H.N., You S.-J., Hosseini-Bandegharaei A., Chao H.-P. (2017). Mistakes and inconsistencies regarding adsorption of contaminants from aqueous solutions: A critical review. Wat. Res..

[41] Dzwigaj S., Massiani P., Davidson A., Che M. (2000). Role of silanol groups in the incorporation of V in β zeolite. J. Mol. Catal. A Chem..

[42] Çelebi O., Üzüm Ç., Shahwan T., Erten H.N. (2007). A radiotracer study of the adsorption behavior of aqueous Ba^2+^ ions on nanoparticles of zero-valent iron. J. Hazard. Mater..

[43] Han R., Zou W., Wang Y., Zhu L. (2007). Removal of uranium(VI) from aqueous solutions by manganese oxide coated zeolite: Discussion of adsorption isotherms and pH effect. J. Environ. Radioactivity.

[44] De Sousa D.N.R., Insa S., Mozeto A.A., Petrovic M., Chaves T.F., Fadini P.S. (2018). Equilibrium and kinetic studies of the adsorption of antibiotics from aqueous solutions onto powdered zeolites. Chemosphere.

[45] Opiso E., Sato T., Yoneda T. (2009). Adsorption and co-precipitation behavior of arsenate, chromate, selenate and boric acid with synthetic allophane-like materials. J. Hazard. Mater..

[46] Pawar R.R., Kim M., Kim J.-G., Hong S.-M., Sawant S.Y., Lee S.M. (2018). Efficient removal of hazardous lead, cadmium, and arsenic from aqueous environment by iron oxide modified clay-activated carbon composite beads. Appl. Clay Sci..

[47] Najafabadi H.H., Irani M., Rad L.R., Haratameh A.H., Haririan I. (2015). Removal of Cu^2+^, Pb^2+^ and Cr^6+^ from aqueous solutions using a chitosan/graphene oxide composite nanofibrous adsorbent. RSC Adv..

[48] Amarasinghe B.M.W.P.K., Williams R.A. (2007). Tea waste as a low cost adsorbent for the removal of Cu and Pb from wastewater. Chem. Eng. J..

[49] WHO (2004). Barium in Drinking-Water.

[50] Jun B.-M., Park C.M., Heo J., Yoon Y. (2020). Adsorption of Ba^2+^ and Sr^2+^ on Ti_3_C_2_T_x_ MXene in model fracking wastewater. J. Environ. Manage..

[51] Mnasri-Ghnimi S., Frini-Srasra N. (2019). Removal of heavy metals from aqueous solutions by adsorption using single and mixed pillared clays. Appl. Clay Sci..

[52] Mahramanlioglu M., Kizilcikli I., Bicer I.O. (2002). Adsorption of fluoride from aqueous solution by acid treated spent bleaching earth. J. Fluorine Chem..

[53] Singha A.S., Guleria A. (2014). Chemical modification of cellulosic biopolymer and its use in removal of heavy metal ions from wastewater. Int. J. Biol. Macromol..

[54] El-Korashy S.A., Elwakeel K.Z., El-Hafeiz A.A. (2016). Fabrication of bentonite/thiourea-formaldehyde composite material for Pb(II), Mn(VII) and Cr(VI) sorption: A combined basic study and industrial application. J. Clean. Prod..

[55] Sajih M., Bryan N.D., Livens F.R., Vaughan D.J., Descostes M., Phrommavanh V., Nos J., Morris K. (2014). Adsorption of radium and barium on goethite and ferrihydrite: A kinetic and surface complexation modelling study. Geochim. Cosmochim. Acta.

[56] Adeyemo A.A., Adeoye I.O., Bello O.S. (2017). Adsorption of dyes using different types of clay: A review. Appl. Water Sci..

[57] Uddin M.K. (2017). A review on the adsorption of heavy metals by clay minerals, with special focus on the past decade. Chem. Eng. J..

[58] Habib M.A., Bockris J.O., Bockris J.O., Conway B.E., Yeager E. (1980). Specific Adsorption of Ions. Comprehensive Treatise of Electrochemistry.

[59] Creton B., Bougeard D., Smirnov K.S., Guilment J., Poncelet O. (2008). Structural Model and Computer Modeling Study of Allophane. J. Phys. Chem. C.

[60] Baerlocher C., McCusker L.B., Olsen D.H. (2007). Atlas of Zeolite Framework Types.

[61] Dove P.M., Nix C.J. (1997). The influence of the alkaline earth cations, magnesium, calcium, and barium on the dissolution kinetics of quartz. Geochim. Cosmochim. Acta.

[62] De Gisi S., Lofrano G., Grassi M., Notarnicola M. (2016). Characteristics and adsorption capacities of low-cost sorbents for wastewater treatment: A review. Sust. Mater. Technol..

[63] Mu W., Du S., Yu Q., Li X., Wei H., Yang Y. (2018). Improving barium ion adsorption on two-dimensional titanium carbide by surface modification. Dalton Trans..

[64] Kapashi E., Kapnisti M., Dafnomili A., Noli F. (2019). Aloe Vera as an effective biosorbent for the removal of thorium and barium from aqueous solutions. J. Radioanal. Nucl. Chem..

[65] Noli F., Kapnisti M., Buema G., Harja M. (2016). Retention of barium and europium radionuclides from aqueous solutions on ash-based sorbents by application of radiochemical techniques. Appl. Radiat. Isot..

[66] Mishra S.P., Singh V.K. (1995). Radiotracer Technique in Adsorption Study-XIII. Adsorption of Barium and Cesium Ions on Chromium(IV) Oxide Powder. Appl. Radiat. Isot..

[67] Mishra S.P., Singh V.K. (1995). Radiotracer Technique in Adsorption Study-XI. Adsorption of Barium and Cesium Ions on Hydrous Ceric Oxide. Appl. Radiat. Isot..

[68] Torab-Mostaedi M., Ghaemi A., Ghassabzadeh H., Ghannadi-Maragheh M. (2011). Removal of strontium and barium from aqueous solutions by adsorption onto expanded Perlite. Can. J Chem. Eng..

[69] Faghihian H., Marageh M.G., Kazemian H. (1999). The use of clinoptilolite and its sodium form for removal of radioactive cesium, and strontium from nuclear wastewater and Pb^2+^, Ni^2+^, Cd^2+^, Ba^2+^ from municipal wastewater. Appl. Radiat. Isot..

[70] Younis S.A., Ghobashy M.M., Bassioni G., Gupta A.K. (2020). Tailored functionalized polymer nanoparticles using gamma radiation for selected adsorption of barium and strontium in oilfield wastewater. Arab. J. Chem..

[71] Kaveeshwar A.R., Kumar P.S., Revellame E.D., Gang D.D., Zappi M.E., Subramaniam R. (2018). Adsorption properties and mechanism of barium (II) and strontium (II) removal from fracking wastewater using pecan shell based activated carbon. J. Clean. Prod..

