# Supplementary material for: Removal of Barium from Solution by Natural and Iron(III) Oxide-Modified Allophane, Beidellite and Zeolite Adsorbents
Source: Materials (Basel). 2020 Jun 5;13(11):2582. doi: 10.3390/ma13112582 (PMC7321624; doi:10.3390/ma13112582)
Supplement: Supplementary file 1 [file materials-13-02582-s001.pdf]

materials

MDPI

1 Supplementary Information

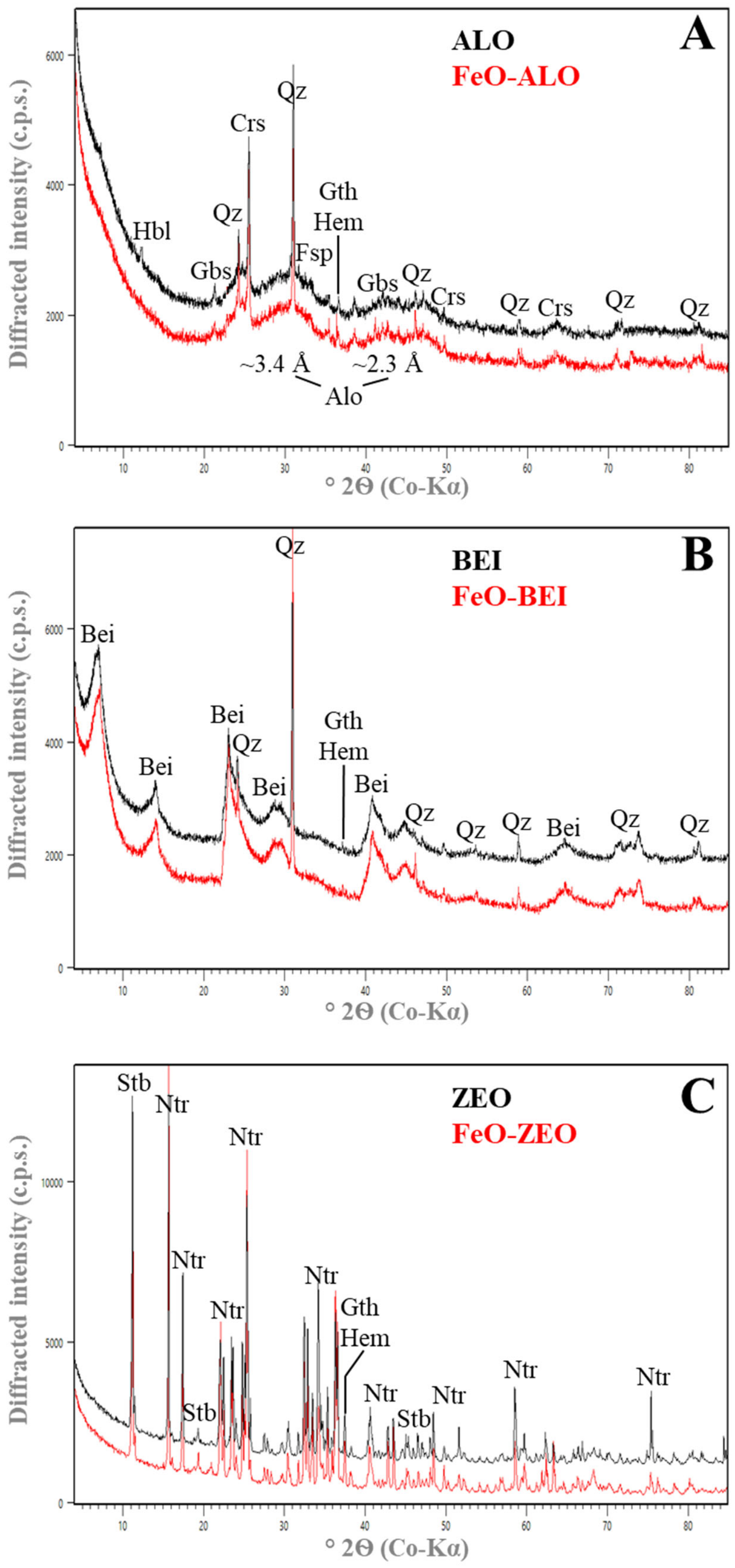

2

3 **Figure S1.** XRD patterns of adsorbents collected after the Ba adsorption.

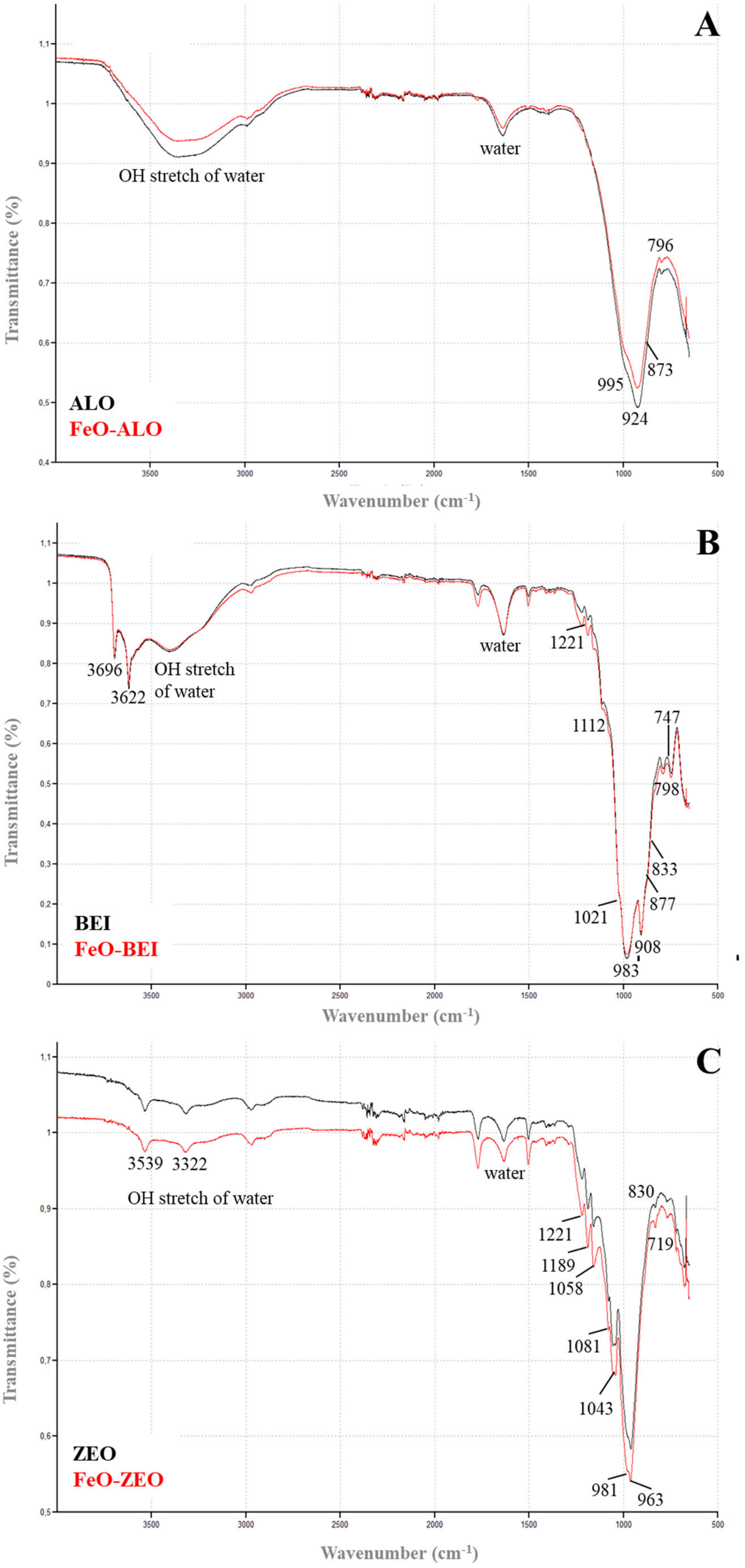

Figure S2. FTIR spectra of adsorbents collected after the Ba adsorption.

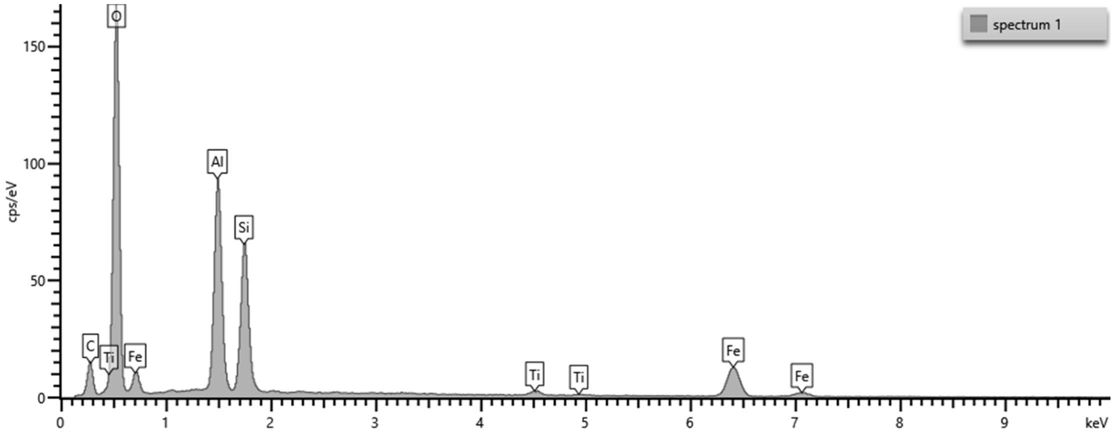
spectrum 1
cps/eV
keV
O
Al
Si
C
Ti
Fe

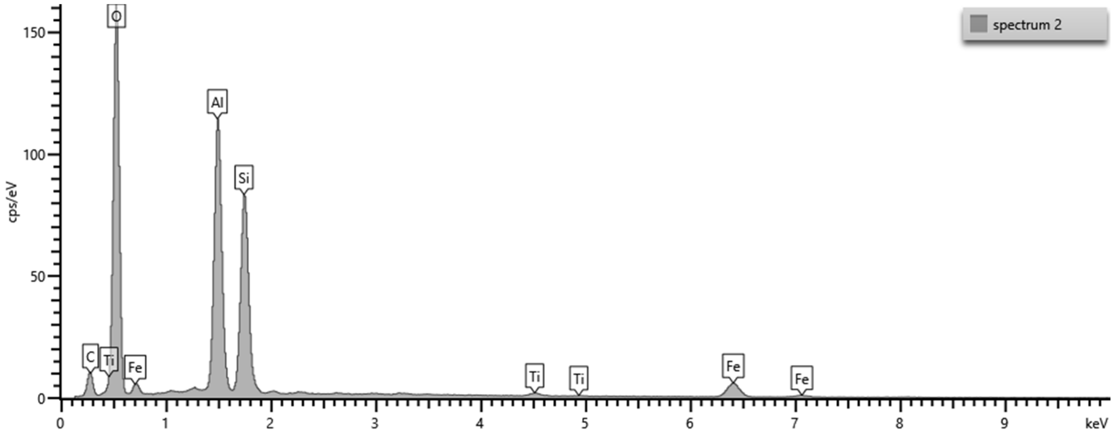
spectrum 2
cps/eV
keV
O
Al
Si
C
Ti
Fe

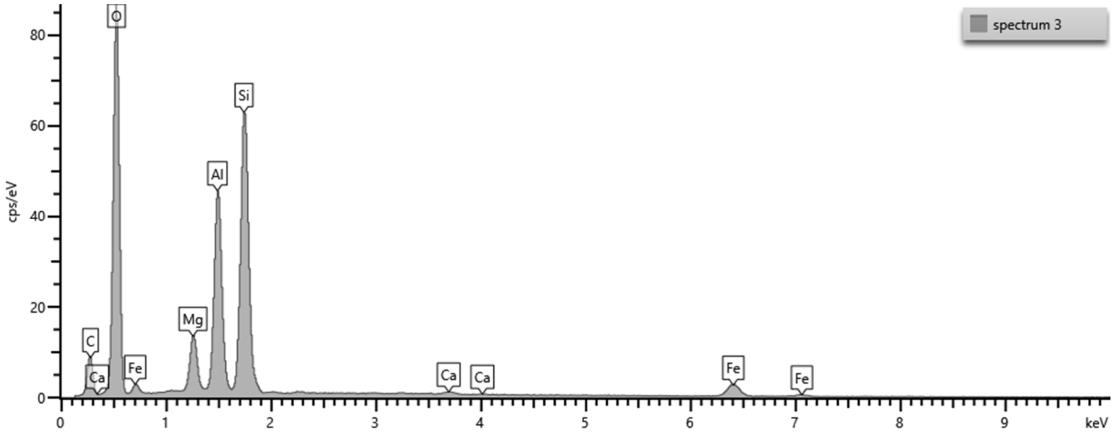
spectrum 3
cps/eV
keV
O
Si
Al
Mg
C
Ca
Fe

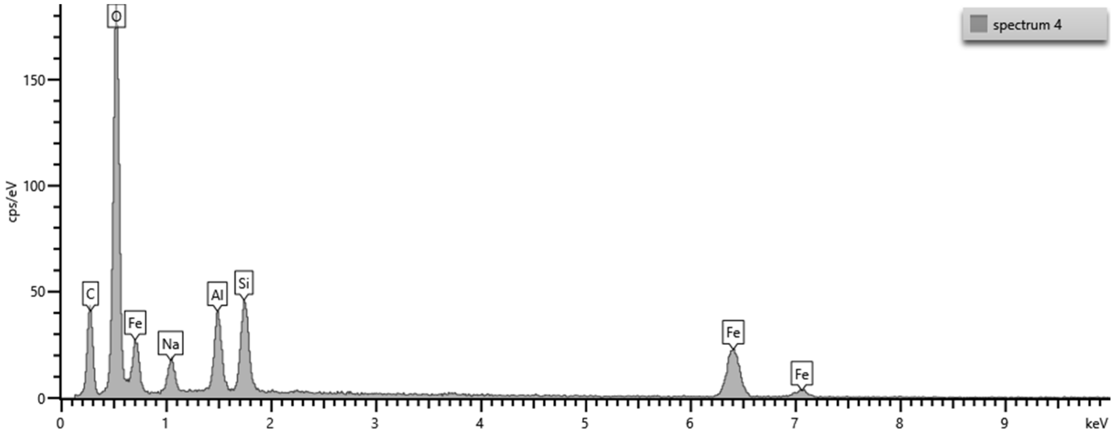
spectrum 4
cps/eV
keV
O
C
Fe
Na
Al
Si

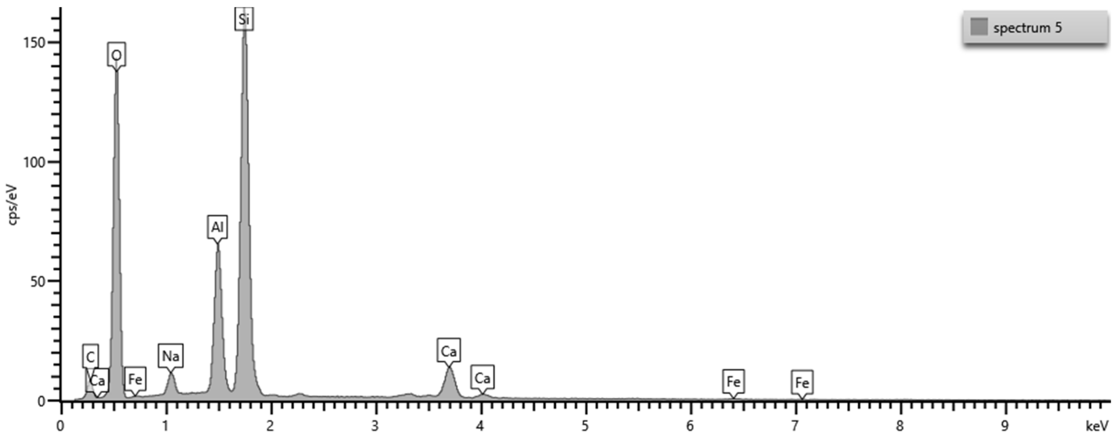

Figure S3. EDX spectra obtained from single spot analyses (areas are marked in Figure 4, third panel).
